# Hemiacetal Ester Side Chains as a Mild Protecting Group for Carboxylic Acids in Polycarbonate Backbones

**DOI:** 10.1002/marc.202500082

**Published:** 2025-02-14

**Authors:** Leon Bixenmann, Lutz Nuhn

**Affiliations:** ^1^ Institute of Functional Materials and Biofabrication Department of Chemistry and Pharmacy Julius‐Maximilians‐Universität Würzburg 97070 Würzburg Germany; ^2^ Max Planck Institute for Polymer Research 55128 Mainz Germany

**Keywords:** biodegradable, hemiacetal ester, polycarbonate, polycarboxylate, protecting group

## Abstract

Hemiacetal esters are versatile functional groups known for their unique ability to degrade under mild conditions such as exposure to water, alcohols, organic acids, or heat. In this study, hemiacetal esters are introduced as mild, transient protecting groups for carboxylic acids along polycarbonate backbones. A six‐membered cyclic carbonate monomer is synthesized by reacting ethyl vinyl ether with a carboxylic acid precursor, demonstrating high efficiency and stability under nucleophilic polymerization conditions. Potential susceptibilities of the hemiacetal esters to transacetalization reactions do not occur under these polymerization conditions. Instead, well‐defined homo and PEG‐based block copolymers are obtained with narrow molecular weight distributions and preserved hemiacetal ester functionalities. These labile side chain groups enabled facile deprotection, yielding polycarbonates with free carboxylic acids, holding significant potential for applications in drug delivery, sustainable polymers, and advanced functional materials.

## Graphical abstract

Hemiacetal esters serve as mild, transient protecting groups for carboxylic acids in polycarbonate backbones. Six‐membered cyclic carbonates with ethyl hemiacetal esters can be polymerized into well‐defined polycarbonates with narrow molecular weight distributions and preserved hemiacetal ester functionalities. They allow for easy deprotection toward potential applications in drug delivery and sustainable materials.

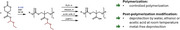

## 1 Introduction

Recent advancements have highlighted the potential of polymers incorporating hemiacetal esters as versatile functional group gaining increasing attention especially due to their uniquely mild degradation profiles by reaction with water,^[^ ^1^, ^2^, ^3^, ^4^, ^5^, ^6^, ^7^, ^8^, ^9^, ^10^ ^]^ alcohols,^[^ ^6^, ^11^, ^12^ ^]^ carboxylic acid,^[^ ^13^, ^14^, ^15^ ^]^ or simple treatment with heat.^[^ ^4^, ^5^, ^14^, ^15^, ^16^, ^17^, ^18^ ^]^ Therefore, this functional group is extensively studied as degradable linkers integrated into polymers^[^ ^2^, ^6^, ^10^, ^19^, ^20^ ^]^ or as carboxylate protecting groups with transient stability,^[^ ^4^, ^21^, ^22^, ^23^, ^24^ ^]^ affording applications ranging from drug delivery,^[^ ^2^, ^10^, ^25^, ^26^, ^27^ ^]^ sustainable polymers,^[^ ^28^, ^29^ ^]^ or lithography.^[^ ^5^ ^]^

As for the majority of the reported examples, hemiacetal esters have been incorporated into the side chains of polymers with carbon‐carbon backbones, predominantly synthesized via radical polymerization methodologies.^[^ ^12^ ^]^ However, such non‐degradable polymer backbones raise concerns in many potential fields of applications, including pharmaceutical delivery approaches or sustainable chemistries. To that respect, especially aliphatic polycarbonates have recently been gaining increasing attention as degradable alternatives hydrolyzing into products of low toxicity such as diols and carbon dioxide.^[^ ^30^, ^31^ ^]^

However, well‐defined aliphatic polycarbonates as well as many other degradable polymers are mainly accessible through nucleophilic chain‐growth polymerization techniques. The integration of hemiacetal esters into such polymer has likely been considered challenging due to their pronounced susceptibility to nucleophilic attack by weak nucleophiles, such as alcohols.^[^ ^6^, ^11^, ^12^ ^]^ Although this presumption appears intuitively reasonable, the stability of hemiacetal esters is strongly dependent on reaction conditions such as the polarity of the solvent, the reaction temperature, and the applied catalysts.^[^ ^12^ ^]^ Notably, they even exhibit substantial resilience under rigorous carb‐anionic polymerization conditions when polymerized in non‐polar solvents like n‐hexane,^[^ ^32^ ^]^ while hydrolyzing rapidly in water without any additional catalyst present.^[^ ^7^, ^8^, ^9^ ^]^

To extend the versatile advantages of hemiacetal esters to polycarbonates we synthesized a six‐membered carbonate monomer by the addition of ethyl vinyl ether to the free carboxylic acid of 5‐methyl‐2‐oxo‐1,3‐dioxane‐5‐carboxylic acid (MTC‐OH). We show that the susceptibility toward transacetalization reactions does not interfere with the ring‐opening polymerization conditions of the aliphatic polycarbonates. We demonstrate the polymerization of this monomer to form both homo and block copolymers, with a detailed analysis of the resulting polymer properties. Especially the ability to synthesize well‐defined block copolymers is highly interesting for applications in the field of drug delivery or other pharmaceutical purposes.

**Figure 1:**
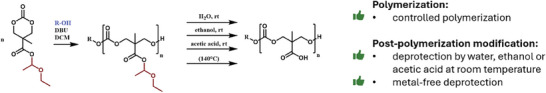
Synthetic approaches to polycarbonates with carboxylate side chains protected as hemiacetal esters prepared from ethyl vinyl ether and overview over their chemical reactivity.

To underline the versatility of this functional group and contribute to the understanding of its chemistry, we particularly focused on their different deprotection conditions behavior. While the developed synthetic approach has not only the potential for developing new aliphatic polycarbonates with hemiacetal esters integrated into their side chain, it also further provides easy synthetic access to aliphatic polycarbonates with free carboxylic acids along their side chains with numerous applications of their own. (**Figure** **1**).

## 2 Results and Discussion

### 2.1 Monomer Synthesis

**Figure 2:**
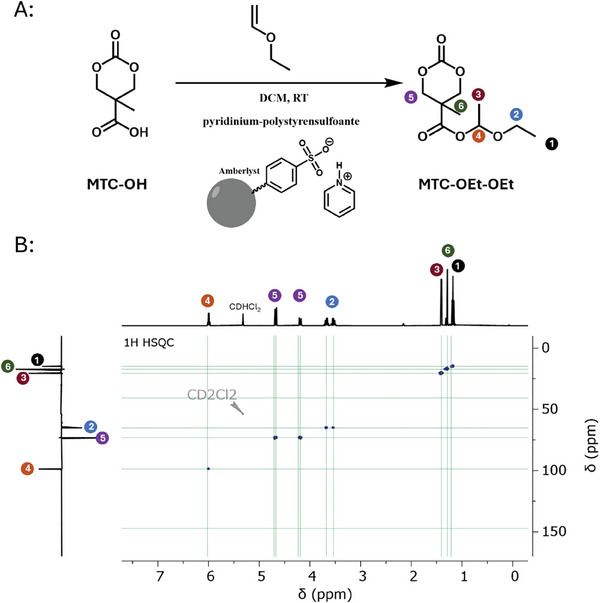
Synthesis of MTC‐OEt‐OEt. A) Monomer synthesis route to six‐membered cyclic carbonate monomers with hemiacetal ester side groups. B) ^1^H ^13^C HSQC NMR spectrum of MTC‐OEt‐OEt.

In general, hemiacetal esters can be conveniently synthesized by the addition of carboxylic acids to vinyl ethers.^[^ ^12^, ^13^, ^15^, ^22^, ^33^, ^34^ ^]^ Therefore, 5‐methyl‐2‐oxo‐1,3‐dioxane‐5‐carboxylic acid (MTC‐OH, **Figure** **2**A) prepared from 3‐hydroxy‐2‐(hydroxymethyl)‐2‐methyl propanoic acid was chosen as an ideal key building block (Figures S1 and S12, Supporting Information), yielding hemiacetal ester monomers in just one additional step. Many vinyl ethers are either commercially available or can be synthesized by one‐step procedures starting from respective alcohols and the addition to acetylene (acetylene can be derived in situ from calcium carbide or 1,2‐dichloroethane).^[^ ^35^, ^36^, ^37^ ^]^ This convergent synthesis strategy is therefore highly attractive as it offers maximum modularity.

Considering the pronounced sensitivity of hemiacetal ester products, we optimized the synthesis by avoiding extensive purifications (including chromatography) while simultaneously enhancing yields and simplifying the synthesis protocols. Acetalization reactions with vinyl ethers typically involve mild catalysts such as phosphoric acids or pyridinium salts. Their low acidity, fortunately, prevents side reactions like both cationic ring‐opening polymerization of the cyclic carbonate and cationic polymerization of the vinyl ether.^[^ ^13^ ^]^ To facilitate the purification process, we selected pyridinium salts of the acidic resin Amberlyst^R^ 15 (H), a commercially available crosslinked polystyrene sulfonic acid (Figure S13, Supporting Information). The solid resin allows for convenient catalyst removal by filtration, while effectively providing catalytic capabilities like pyridinium *p*‐toluene sulfonic acid. Moreover, reaction monitoring can directly be followed visually as the initially insoluble acid MTC‐OH becomes soluble over time in dichloromethane as hemiacetal ester. Access of vinyl ethers was applied to assure quantitative MTC‐OH conversion, which is readily removed by distillation afterward. The obtained hemiacetal ester, in turn, was then collected with sufficient purity for polymerization. The monomer's composition and purity were verified by ^1^H, ^13^C, and 2D‐NMR spectroscopy, with the characteristic hemiacetal ester signal appearing as a quartet at 6.0 ppm (Figure 2B; Figures S8–S23, Supporting Information).

### 2.2 Homopolymerization of Carbonate Monomers with Hemiacetal Ester Side Groups

**Figure 3:**
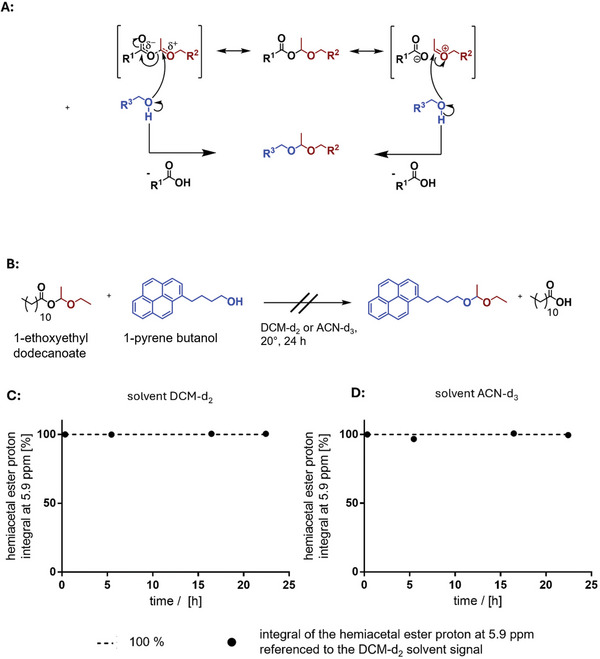
Transacetalization of hemiacetal esters with alcohols. A) The suggestion of a theoretic mechanisms of transacetalization without catalysts. B) Monitoring the transacetalization of 1‐ethoxyethyl decanoate with 1‐pyrene butanol to the corresponding acetal followed by ^1^H NMR spectroscopy in CD_2_Cl_2_ (C) or ACN‐d_3_ D) as solvent.

Despite considerable interest in incorporating hemiacetal esters into polymers, their integration into polymers synthesized via nucleophilic chain‐growth polymerization mechanisms are rare.^[^ ^12^ ^]^ We assume that this has been considered as challenging due to literature reports highlighting their high susceptibility to nucleophilic attacks, even by weak nucleophiles, in the absence of additional catalysts.^[^ ^6^, ^11^ ^]^ For instance, Galluci et al. reported that 1‐(2‐phenoxyethoxy)ethyl acetate reacts rapidly with 2‐phenoxyethan‐1‐ol in chloroform (0.3 m), achieving 95% conversion to the acetal within 30 min.^[^ ^11^ ^]^ Such transacetalizations involving the polymer end group could lead to termination reactions, effectively hindering the polymerization. While the exact mechanism of such transacetalization has to the best of our knowledge not yet been studied more in detail, based on our prior experience, we did not anticipate such rapid transacetalization for most hemiacetal esters in non‐polar aprotic solvents in the absence of acidic catalysts. Possible mechanisms would involve highly charged intermediates or transition states, which usually require conditions stabilizing those intermediates (**Figure** **3**A).

Therefore, to investigate whether a polymerization of cyclic carbonate monomers with hemiacetal esters integrated into the side chain is theoretically feasible, we first synthesized 1‐ethoxyethyl dodecanoate as a sterically unhindered hemiacetal ester model compound (Figures S14–S16, Supporting Information). This compound was incubated at ≈1:1 molar ratio at high concentrations (0.5 m) with pyrene butanol in dichloromethane monitoring the stability of the hemiacetal ester proton at 5.9 ppm (Figure 3B,C).

To ensure the removal of any acidic impurities, we pre‐treated the hemiacetal ester solution by stirring it over calcium hydride in DCM before introducing the alcohol. Contrary to earlier reports of rapid transacetalization of hemiacetal esters in chloroform,^[^ ^6^, ^11^ ^]^ we observed for 1‐ethoxyethyl dodecanoate in dichloromethane no reaction. We were then interested in weather such transacetalization could be observed in more polar solvents, which might further stabilize potential charged intermediates expected during the mechanism of transacetalization^[^ ^8^, ^9^ ^]^ (Figure 3A) and therefore we repeated the experiment in acetonitrile (Figure 3B,D). Despite the increased solvent polarity, no evidence of transacetalization was observed. These findings demonstrate that in aprotic solvents the studied hemiacetal ester group is relatively stable at room temperature in the presence of alcohols when no acidic catalyst is present. Although structural differences between the hemiacetalesters and alcohols could have led to the previously observed unique fast transacetalization, we assume that acidic impurities either introduced by the solvent itself or by already hydrolyzed hemiacetal esters might have led to the reported conclusions.^[^ ^6^, ^11^ ^]^

Confident in the sufficient stability of the hemiacetal ester moiety, we proceeded with the nucleophilic ring‐opening polymerization of MTC‐OEt‐OEt as monomer. Polycarbonates can generally be synthesized under both acidic and base‐catalyzed polymerization conditions. However, under acidic conditions, side reactions involving the hemiacetal ester side chain are difficult to avoid.^[^ ^12^ ^]^ Therefore, we employed base‐catalyzed conditions, applying mild reaction conditions previously established for the ring‐opening polymerization of cyclic carbonates.^[^ ^38^, ^39^, ^40^ ^]^ Special care was taken to quench any residual water or acidic impurities by stirring the monomer stock solution over calcium hydride for at least three days. Reactions were conducted at −20 °C in dichloromethane at a monomer concentration of 0.5 m with of DBU as the catalyst (Figure 3A; Figure S25, Supporting Information).

**Figure 4:**
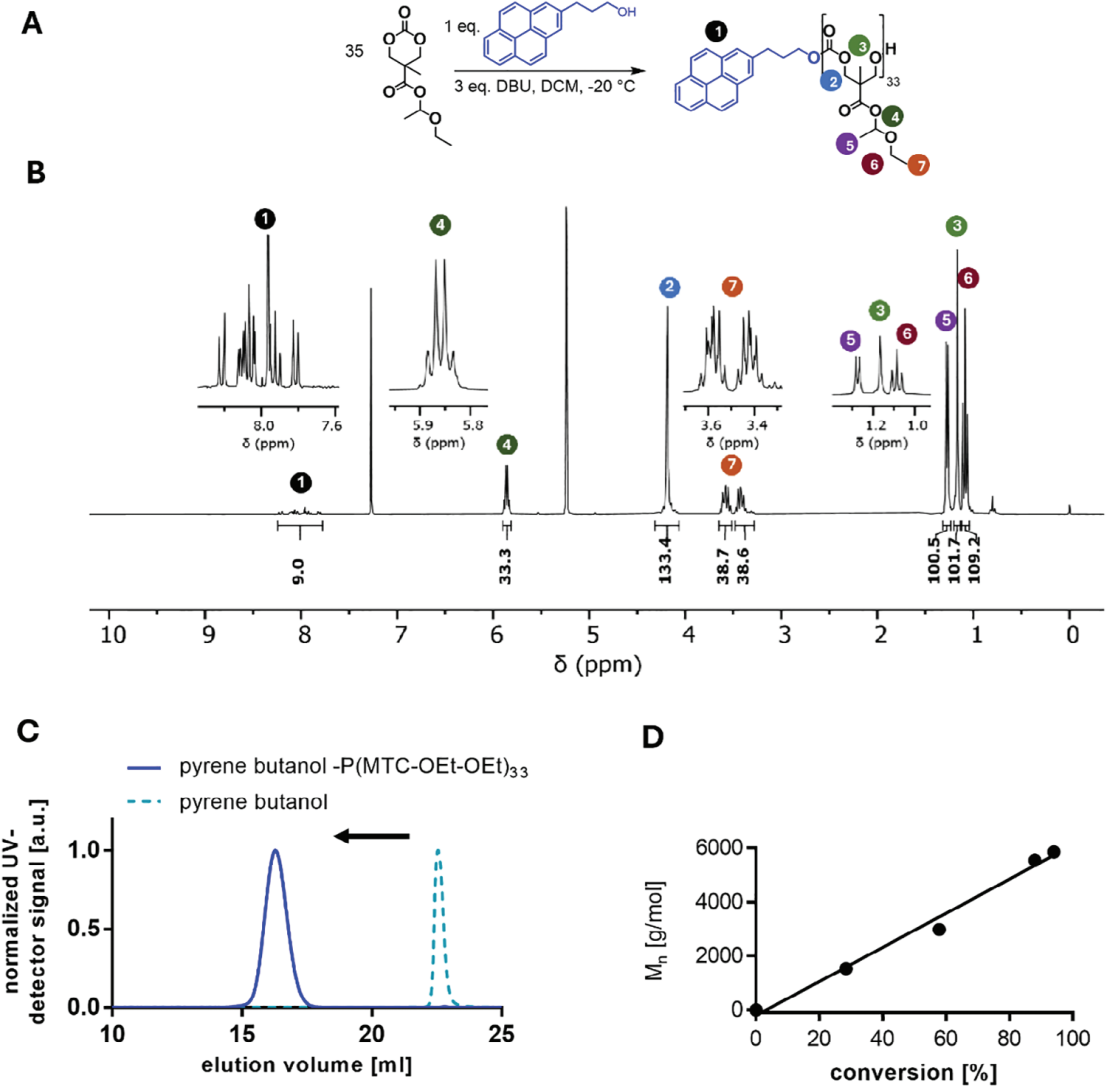
Synthesis and characterization of pyrene butanol‐initiated homopolymer P(MTC‐OEt‐OEt)_33_. A) Synthesis scheme for the polymerization with DBU as a basic catalyst. B) ^1^H NMR spectra of the polymer with characteristic proton signals at ≈5.9 ppm (proton number 4) proving the preservation of the hemiacetal ester moiety under the polymerization conditions. C) Size exclusion chromatogram (SEC) of pyrene butanol‐P(MTC‐OEt‐OEt) compared to the initiator pyrene butanol recorded by absorbance at 344 nm (for RI signal see Figure S29, Supporting Information). D) Evolution of molecular weight (THF SEC (RI signal), referenced to PMMA standards) with respect to monomer conversion (determined by the shift of proton 2 (Figure 4A) in the ^1^H NMR spectrum).

In case of potential side reactions, particularly the potential transacetalization of the polymer end group, we would expect termination reactions diminishing the overall rate of polymerization. Therefore, we followed the evolution of molecular weight determined by size exclusion chromatography (SEC) with respect to monomer conversion determined by ^1^H NMR. As shown in **Figure** **4**D, the molecular weight determined by SEC increases linearly with conversion, indicating controlled polymerization conditions with uniform initiation and absence of major termination or transfer reactions (Figure 4D; Table S1, Supporting Information).

As initiator, we used pyrene butanol, which compared to the monomer absorbs UV light at 344 nm. Consequently, successful initiation can be readily confirmed by SEC recording the absorbance at 344 nm wavelength (Figure 4D). Again, in case of major termination reactions or even initial acetalization of the alcohol initiator, we would expect a tailing or even remaining non‐initiated pyrene butanol. On the contrary, a drastic shift of the pyrene butanol initiator signal could be recorded and proves the absence of any non‐converted pyrene butanol. Furthermore, based on the monomodal SEC UV‐ (Figure 4D) and RI‐recorded elugram (Figure S29, Supporting Information), affording narrow polymer dispersities of (Đ ≈ 1.1, Table S2, Supporting Information), other polymer formation by monomer self‐initiation (for instance by water) without pyrene butanol can further be successfully excluded under the applied polymerization conditions.

Notably, these conditions also effectively preserve the labile hemiacetal ester bond in the polymer side chain, as confirmed by the characteristic proton signal ≈5.9 ppm observed in the ^1^H NMR spectra (Figure 4B; Figures S26–S28, Supporting Information). Furthermore, referencing the aromatic pyrene signal to nine protons, a monomer repeating unit was determined at 33, which matches with the expected polymer length at a monomer conversion of ≈95% for a monomer to initiator ratio of 35:1 (Figure 4A,C; Table S2, Supporting Information).

Unfortunately, subsequent MALDI‐TOF mass spectrometry measurements did not provide sufficient of adequate quality to be analyzed, probably as a result of the labile hemiacetal ester bond already degrading under the applied sample preparation methods. In accordance with this, after complete deprotection of the hemiacetal ester side chains, a well‐defined polymer mass distribution with distinct peak signals should still be available for such samples (as confirmed later on in Figure 6E,F).

To further confirm the absence of transacetalization during polymerization, we incubated the monomer MTC‐OEt‐OEt under basic conditions (0.5 m concentration in DCM) for 6 h at room temperature, simulating the basic polymerization conditions by adding DIPEA (DIPEA itself cannot efficiently catalyze the polymerization) (Figure S24A, Supporting Information). Pyrene butanol was used as a model alcohol initiator and benzyl 3‐hydroxy‐2‐(hydroxymethyl)‐2‐methylpropanoate (bisMPA‐Bn) as a model polymer alcohol end group at a 1:1 (monomer: alcohol functionality) molar ratio. Even under these much harsher conditions, which exceed those encountered during the actual polymerization, no significant rates of acetalization were observed (see Figure S24B,C, Supporting Information).

### 2.3 Block Copolymerization of Amphiphilic Polycarbonates with Hemiacetal Ester Side Chains

**Figure 5:**
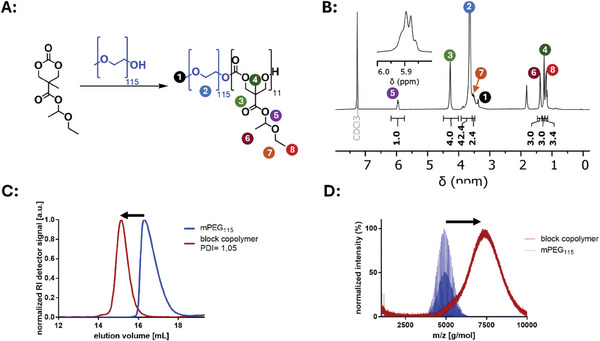
Synthesis and characterization of the block copolymer mPEG_115_‐*b*‐P(MTC‐OEt‐OEt)_14._ A) The polymerization was conducted at −20 °C in DCM, initiated by the macroinitiator mPEG_115_ and catalyzed by the organic base DBU. B) ^1^H NMR characterization of the block copolymer. C) THF SEC trace analysis and D) MALDI TOF MS analysis of the block copolymer compared to the mPEG_5k_‐OH macroinitiator (solvent DCM, matrix: DCTB supplemented with Na TFA).

The DBU‐catalyzed ring opening polymerization conditions could also successfully be applied to the formation of block copolymers with mPEG_5k_‐OH as macroinitiators (**Figure** **5**A).^[^ ^38^, ^39^, ^41^ ^]^ PEG‐based polycarbonate block copolymers are especially interesting for various biomedical applications.^[^ ^38^, ^41^ ^]^ In accordance with the homopolymerization, the ^1^H NMR spectroscopy analysis of the block copolymerization confirmed the preservation of the hemiacetal esters in the side chain (Figure 5B; Figure S28, Supporting Information). We observed a distinct shift of the resulting block copolymer by SEC and MALDI‐TOF MS relative to the mPEGOH macroinitiator, demonstrating successful polymer block formation (Figure 5C,D; Table S1 and Figures S30 and S32, Supporting Information).

### 2.4 Polymer Deprotection

Hemiacetal esters are, with some exceptions, under specific conditions prone to transacetalization reactions with alcohols,^[^ ^6^, ^11^, ^12^ ^]^ but they also react with water^[^ ^1^, ^2^, ^3^, ^4^, ^5^, ^6^, ^7^, ^8^, ^9^, ^10^, ^12^ ^]^ and carboxylic acid.^[^ ^12^, ^13^, ^14^, ^15^ ^]^ Furthermore, by thermal treatment the hemiacetal esters dissociate to vinyl ethers and carboxylic acids easily.^[^ ^11^, ^12^, ^42^ ^]^ Several studies have reported that in highly polar protic solvents (including water), the intermediate oxocarbenium ion is sufficiently stabilized to enable spontaneous transacetalization (compare Figure 3A), resulting in pH‐independent hydrolysis profiles for the hemiacetal esters.^[^ ^7^, ^8^, ^9^ ^]^ This pronounced sensitivity provides access to exceptionally mild deprotection protocols, which are challenging to achieve with other functional groups.^[^ ^43^, ^44^, ^45^, ^46^, ^47^, ^48^, ^49^, ^50^ ^]^ For instance, for previous efforts to synthesize polycarbonates with free carboxylic acid side chains, monomers were polymerized with protecting groups such as *tert*‐butyl or benzyl esters. However, these approaches require for later deprotections strong acidic or reductive conditions, limiting the versatility of other functional groups incorporated into those polymers.^[^ ^43^, ^44^, ^45^, ^46^, ^47^, ^48^, ^49^, ^50^ ^]^ To demonstrate the versatile reactivity of the hemiacetal ester side chains and their potential as mild protecting group for carboxylic acids in polycarbonates, we deprotected the polymer side chains by treatment with ethanol, water, acetic acid or simply by heat and detailly analyzed the resulting polymers.

While no significant transacetalization was observed in aprotic solvents under conditions employed during polymerization (Figure 3B–D), we were particularly interested whether ethanol could efficiently deprotect the hemiacetal ester moiety when used as both protic solvent and reactant. Deprotection using ethanol is particularly appealing due to its benign nature and the fact that it produces only dialkoxy‐acetals as side‐products, making it an efficient option for mild deprotection.

**Figure 6:**
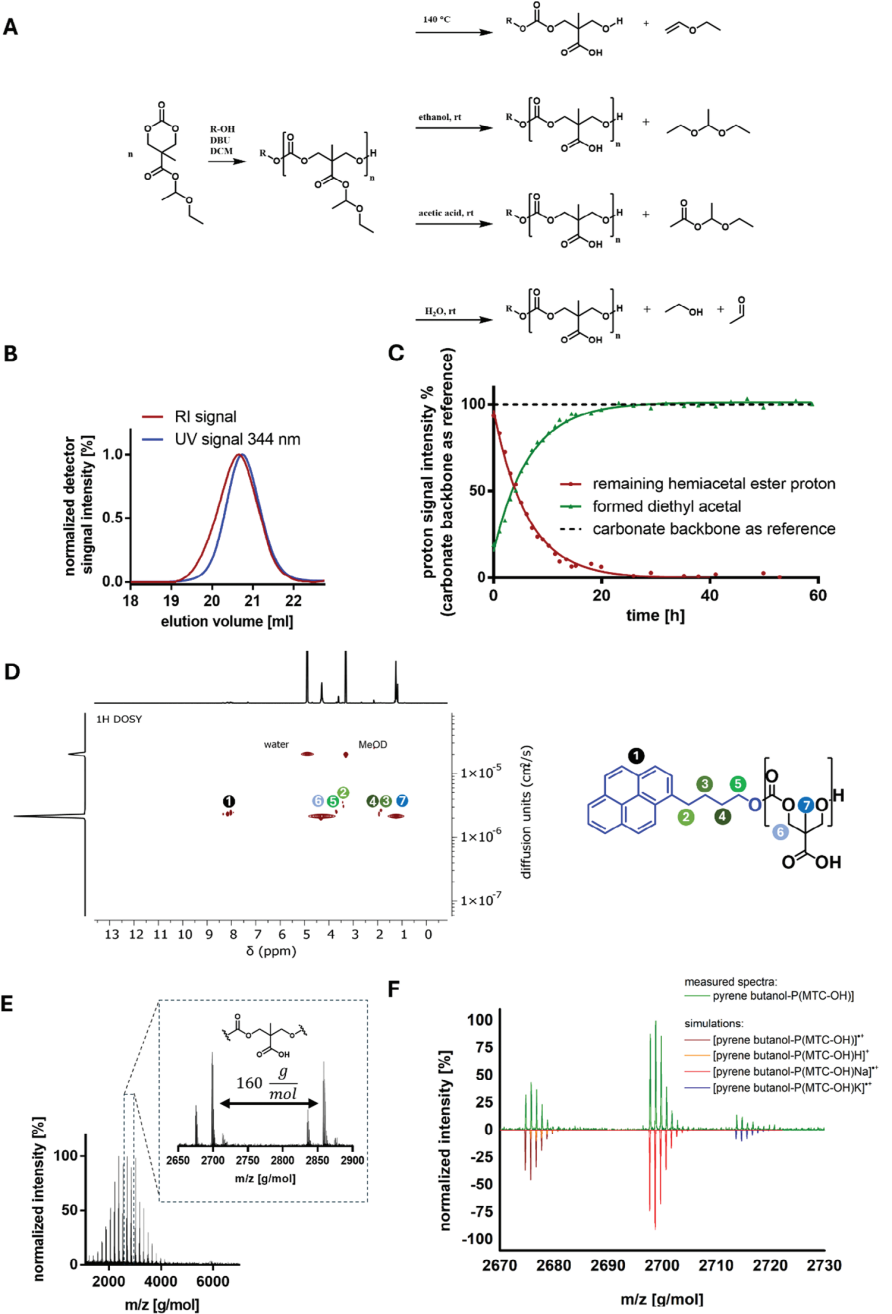
Post‐polymerization hemiacetal ester deprotection. A) Deprotection can be achieved through thermal treatment or by dissolving the polymer in ethanol, acetic acid, or aqueous solutions under neutral and acidic pH conditions. B) HFIP‐SEC elugram of pyrene butanol‐P(MTC‐OH)_33_ obtained by deprotection with ethanol‐d_6_. C) Kinetic ^1^H NMR spectroscopic analysis of hemiacetal ester deprotection by dissolving the homopolymer pyrene butanol‐P(MTC‐OEt‐OEt)_33_ in deuterated ethanol and D) ^1^H DOSY measurement of pyrene butanol‐P(MTC‐OH)_33_ (MestReNova 12.04, method: Peak Heights Fit, Points of diffusion: 128). E) MALDI‐TOF MS analysis of pyrene butanol‐P(MTC‐OH)_33_ (solvent: water, matrix: HCCA with KTFA) F) Overlay of the recorded MALDI‐TOF spectrum (green, shown in the positive direction) and simulations of polymer species pyrene butanol‐P(MTC‐OH) ionized as radical cation (brown), ionized by proton (orange), sodium ion (red) and potassium ion (blue) shown in the negative direction.

To follow the deprotection of the hemiacetal ester side chain, we dissolved the polymer in deuterated ethanol and recorded ^1^H NMR spectra sequentially. As can be seen in **Figure** **6**C (and Figure S34, Supporting Information) the polymer is fully deprotected after 24 h at room temperature. The formed diethyl acetal can easily be removed together with ethanol by subsequent distillation. The residual polymer is obtained as pure material without detectable side reactions at the polymer backbone, according to ^1^H NMR spectroscopy, including DOSY measurements, and SEC trace analyses (Figure 6B–D; Figure S33, Supporting Information).

Alternatively, hemiacetal esters are also well‐known for their thermal instability. Thermal deprotection of functional groups as polymer post‐polymerization processing is highly advantageous as it can even be conducted in bulk without any solvent (note that polymers often suffer from low solvent solubilities).^[^ ^12^, ^16^, ^23^, ^24^, ^42^ ^]^ For example, this thermal lability has been used to increase the shelf‐life of epoxy formulations by deprotecting hemiacetal esters into carboxylic acids on demand by a simple treatment with heat.^[^ ^23^, ^24^ ^]^ Furthermore, during thermal (anhydrous) hemiacetal ester degradation, the respective vinyl ethers are directly liberated.^[^ ^16^, ^18^, ^24^, ^42^ ^]^ This would potentially enable a closed‐loop process to use them again for monomer protection and making the process highly interesting as sustainable alternative procedure.

To test this thermal deprotection process, we performed thermogravimetric analyses of the polymer (Figure S35, Supporting Information). In accordance with literature reports,^[^ ^13^ ^]^ initial degradation occurred already at 140 °C (unfortunately, a defined thermolysis of only hemiacetal esters could not be recorded under the applied temperature gradients, as it gradually merged with carbonate backbone degradation).^[^ ^16^, ^17^, ^28^, ^51^ ^]^ We therefore applied a constant temperature of 140 °C to the solid polymer sample and recorded its weight loss by TGA for 120 min (Figure S36, Supporting Information). Here, TGA analysis showed an initial fast mass loss due to hemiacetal ester side chain degradation followed by a minimal but steady mass loss attributed to polymer backbone degradation (Figures S36 and S37, Supporting Information). Subsequent ^1^H NMR analysis confirmed a quantitative loss of the hemiacetal ester signals, while the proton signal of the polycarbonate backbone remained almost unaltered (Figure S39, Supporting Information). Unfortunately, SEC trace in HFIP as well as in THF revealed a massive alteration of the polymer's total molecular weight and broadened dispersities probably arising from backbone side reactions including backbiting and chain transfer reactions (Figure S38, Supporting Information). Nonetheless, deprotecting hemiacetal esters thermally at rather low temperatures might be still a valuable method for thermally more resistant polycarbonates.

**Figure 7:**
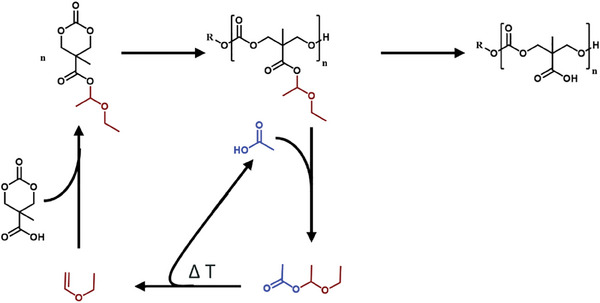
Proposed closed‐loop‐process for carboxylic acid protection installed as to cyclic carbonate monomers and their deprotection from polycarbonate side chains. The monomer is synthesized by addition of the free acid to vinyl ethers forming and hemiacetal ester bond. After polymerization, the carboxylic acid is deprotected by transacetalization with an excess of acetic acid. The formed small molecular hemiacetal ester could then serve as a precursor for vinyl ether synthesis by thermolysis as reported by literature.^[^ ^42^ ^]^

Instead of thermal deprotection and recycling of the liberated vinyl ethers, hemiacetal esters can also be conventionally transferred to other organic acids by transacetalization at room temperature.^[^ ^11^, ^12^, ^13^ ^]^ This deprotection step allows for mild deprotection of the polymer, too, while the liberated hemiacetal ester might again potentially serve as a valuable precursor for new monomer protection (**Figure** **7**). This would in principle result in a closed‐loop process, circumventing direct thermal treatment of the polymer.

For that purpose, pyrene butanol–P(MTC‐OEt‐OEt)_33_ was dissolved in pure acetic acid and stirred for 5 h. After subsequent removal of acetic acid by distillation, the pure deprotected polymer was obtained and confirmed by ^1^H NMR spectroscopy (Figure S40, Supporting Information) and SEC (Figure S41 and Table S6, Supporting Information). MALDI TOF spectrometry further provided proof that under these conditions the polymer backbone is not affected (Figure 5E+F), which allows an additional straightforward access to carboxylate‐functionalized Poly(carbonates).

Despite the size discrepancy between the obtained degree of polymerization determined by ^1^H NMR and the observed molecular weight distribution observed by MALDI‐TOF MS, we notably found only species which could solely be attributed to the structure of the fully deprotected P(MTC‐OEt‐OEt) with pyrene butanol end group (Figure 6F). Further polymers initiated by water, ethanol, or cyclic structures which could arise due to premature hydrolysis, could not be observed (Figure S42, Supporting Information). Therefore, due to these otherwise very conclusive evidences, we assume that mass discrimination effects either during the measurement or the sample preparation might be responsible for the observed discrepancy in degree of polymerization.

Finally, we were also interested whether a direct hydrolytic treatment with water can also efficiently cleave the hemiacetal ester bond.^[^ ^12^ ^]^ Water as a deprotecting agent is especially beneficial as it is environmentally friendly and allows large scale processing. The released ethanol and acetaldehyde are also water‐soluble and can easily be removed. They are considered biodegradable which may promote the production process, too.

**Figure 8:**
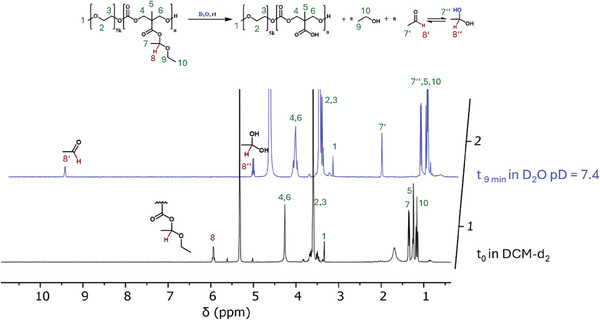
Hydrolysis followed by ^1^H NMR spectroscopy of mPEG_5k_‐*b*‐P(MTC‐OEt‐OEt)_11_ in neutral 0.4 m phosphate buffered solution.

While a direct deprotection of pyrene butanol‐P(MTC‐OEt‐OEt) homopolymer in water at room temperature is impeded by its low water solubility, we selected the synthesized amphiphilic mPEG_5k_‐*b*‐P(MTC‐OEt‐OEt) block copolymer for the hydrolytic hemiacetal ester deprotection. The polymer was dissolved in water at both neutral and acidic conditions readily, enabling an efficient deprotection process. Notably, when we aimed at monitoring the hydrolysis over time, we observed already a full deprotection by the first recorded ^1^H‐NMR measurement after ≈9 min underlining the high susceptibility toward rapid hydrolysis of the hemiacetal esters (**Figure** **8**). Nonetheless, the water treatment must not necessarily be performed for such short reaction times, as after 5 h the backbone of the deprotected polymer still remained fully intact, as characterized by ^1^H NMR spectroscopy and SEC analysis (Figures S43–S48, Supporting Information). In the ^1^H NMR spectrum, the hemiacetal ester signals were as expected fully absent and in the SEC trace the deprotected polymer backbone remained monomodal with narrow dispersities.

## 3 Conclusion

In conclusion, we successfully introduced hemiacetal esters as functional groups for the synthesis of aliphatic polycarbonates, demonstrating their stability under the applied polymerization conditions. We showed that transacetalization, a common concern for hemiacetal esters, can be effectively mitigated in aprotic non‐polar solvents. We are convinced this will pave the way for the successful combination of hemiacetal ester side chains with various other aliphatic polycarbonate backbones and potentially even other classes of degradable polymers. Furthermore, we performed a detailed analysis of different hemiacetal ester degradation conditions, which may contribute to a better understanding of their chemistry in general. To that respect, the rather transient stability of the studied hemiacetal ester prepared from ethyl vinyl ether makes them ideal protecting groups in polymer chemistry, offering facile deprotection under mild conditions, e.g. hydrolysis, alcoholysis, acidolysis or thermolysis. We expect that the developed preparative method may encourage further research and chemical diversification of hemiacetal esters offering access to derivatives with increased stability that may become relevant for drug delivery purposes. Perspectively, hemiacetal esters may not only serve as protecting group for carboxylates, but also for alcohols or aldehydes.

## Conflict of Interest

The authors declare no conflict of interest.

## Supporting information



Supporting Information

## Data Availability

The data that support the findings of this study are available in the supplementary material of this article.
