# Supplementary material for: Hemiacetal Ester Side Chains as a Mild Protecting Group for Carboxylic Acids in Polycarbonate Backbones
Source: Macromol Rapid Commun. 2025 Feb 14;46(9):2500082. doi: 10.1002/marc.202500082 (PMC12051728; doi:10.1002/marc.202500082)
Supplement: Supplementary file 1 — Supporting Information [file MARC-46-2500082-s001.pdf]

Macromolecular Rapid Communications

Supporting Information

for *Macromol. Rapid Commun.*, DOI 10.1002/marc.202500082

Hemiacetal Ester Side Chains as a Mild Protecting Group for Carboxylic Acids in Polycarbonate Backbones

*Leon Bixenmann and Lutz Nuhn**

Hemiacetal ester side chains as a mild protecting group for carboxylic acids in polycarbonate backbones

Leon Bixenmann[1,2], Lutz Nuhn[1,2*]

[1] Institute of Functional Materials and Biofabrication, Department of Chemistry and Pharmacy, Julius-Maximilians-Universität Würzburg, 97070 Würzburg, Germany

[2] Max Planck Institute for Polymer Research, 55128 Mainz, Germany

*corresponding author Prof. Dr. Lutz Nuhn (lutz.nuhn@uni-wuerzburg.de, lutz.nuhn@mpip-mainz.mpg.de)

Supporting Information:

Materials **2**

Instrumentation **2**

Synthetic procedures **4**

Synthesis of benzyl 3-hydroxy-2-(hydroxymethyl)-2-methylpropanoate (B) 4

Synthesis of benzyl 5-methyl-2-oxo-1,3-dioxane-5-carboxylate (C) 7

Synthesis of 5-methyl-2-oxo-1,3-dioxane-5-carboxylic acid (D) 9

Synthesis of the pyridinium-P(styrene sulfonate) solid phase catalyst: 11

Synthesis of 1-ethoxyethyl dodecanoate 12

Synthesis of hemiacetal ester functionalized six-membered carbonate monomers. 14

Polymerizations **18**

Kinetic analysis of the hemiacetal ester stability in presence of alcohols. 18

Homopolymerization 20

Block Copolymerization 24

Hemiacetal ester deprotection **27**

Deprotection by ethanol: 27

Hemiacetal ester deprotection by thermolysis: 30

Deprotection by acetic acid: 34

Aqueous Block Copolymer Deprotection: 37

SI References **42**

Experimental Section

Materials

All purchased solvents and reagents were used without further purification. Hexafluoro isopropanol (HFIP) for SEC trace analysis was purchased from Fluorochem LTd. and distilled for reuse. For general filtration purposes, including the removal of calcium hydride and the preparation of samples for size exclusion chromatography, Fisherbrand™ PTFE filters with a pore size of 0.2 µm were used.

Instrumentation

Size exclusion chromatography. Analytical size exclusion chromatography (SEC) was carried out using THF or HFIP as the eluent.

Measurements with THF as eluent were conducted at a flow rate of 1.0 mL/min and a temperature of 30 °C. The column material utilized was styrene divinylbenzene (particle size of 3 µm, porosity 1000 Å +1000 Å), obtained from Polymer Standards Service GmbH in Mainz, Germany. Detection included both IR-detection (Agilent 1260 Infinity RID) and UV-detection (Agilent 1260 Infinity VWD). As reference molecular weight standards, PMMA was used for calibrating the RI as well as the UV-detector signal at 254 nm (purchased from PSS Polymer Standards Services GmbH). All samples were filtered through a PTFE filter with a pore size of 0.2 µm before analysis.

Analytical SEC with HFIP as eluent were carried out at 40°C. HFIP was supplemented with 3g/L potassium trifluoroacetate. The flow rate was set at 0.8 mL/min. PFG columns purchased from PSS Polymer Standards Service GmbH were used. Those columns are composed of polar-modified silica-based particles (particle size 7 µm, porosity 100 Å + 1000 Å). The setup was equipped with SECurity2isocratic pump, a degasser, an autosampler a column thermostat an RI, and a variable wavelength detector. As reference molecular weight standards, PMMA was used for calibrating the RI as well as the UV-detector signal at 254 nm (PSS Polymer Standards Services GmbH). All samples were filtered through a PTFE filter with a pore size of 0.2 µm before analysis.

Nuclear magnetic resonance spectroscopy. NMR spectra were recorded on a Bruker Avance III 250 MHz Bruker, Avance III 300 MHz, a Bruker Avance Neo 400, and a Bruker Avance III 700 MHz Samples were prepared using deuterated solvents obtained from Sigma-Aldrich. Spectra were analysed using MestReNova 14.2.0 and 12.0.4 by Mestrelab Research and TopSpin® 4.4.0 provided by Bruker.

Thermogravimetric analysis (TGA):

Thermogravimetric analysis was conducted using a TG 209 F1 IRIS (Netzsch, Selb, Germany). Samples (10 - 20 mg) were placed in aluminum oxide crucibles (Netzsch) and heated under ambient air, with mass loss being recorded. The specific heating parameters are detailed in the supporting information below the experimental results.

MALDI-TOF MS.

Matrix-Assisted Laser Desorption/Ionization-Time of Flight Mass Spectrometry (MALDI-TOF MS) was performed using a rapifleX™ MALDI-TOF/TOF mass spectrometer (Bruker Daltonik GmbH, Fahrenheitstraße 4, 28359 Bremen). The instrument is equipped with a scanning smartbeam 10 kHz Nd:YAG laser operating at a wavelength of 355 nm and a 10-bit 5 GHz digitizer. Measurements were conducted in positive ion mode. Calibration was performed using polyethylene glycol polymer standards. Samples of pyrene butanol-P(MTC-OH)$_{33}$ were prepared in water with α-Cyano-4-hydroxycinnamic acid (HCCA) as the matrix supplemented with potassium trifluoroacetate. Samples of mPEG$_{5k}$-P(MTC-OEt-OEt)$_{33}$ were prepared in DCM with 2-[(2E)-3-(4-tert-butylphenyl)-2-methylprop-2-enylidene]malononitrile (DCTB) as the matrix supplemented with sodium trifluoroacetate. Data analysis was carried out using mMass version and plotted in GraphPad Prism 6.

Synthetic procedures

Synthesis of benzyl 3-hydroxy-2-(hydroxymethyl)-2-methylpropanoate (B)

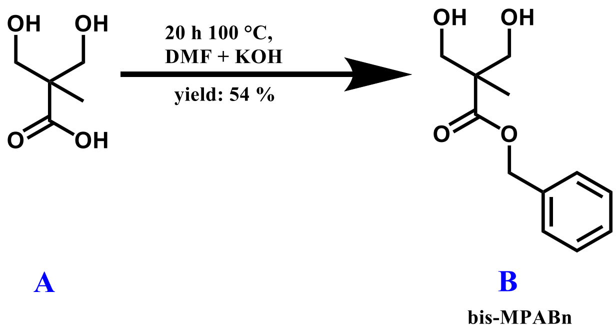

Figure S1: Synthesis of benzyl 3-hydroxy-2-(hydroxymethyl)-2-methylpropanoate abbreviated as BisMPA-Bn

Synthesis of benzyl 3-hydroxy-2-(hydroxymethyl)-2-methylpropanoate (bisMPA-Bn), Figure S1. Benzyl 3-hydroxy-2-(hydroxymethyl)-2-methylpropanoate was synthesized following an adapted literature procedure.[1–3] 45.0 g (335.5 mmol, 1.0 eq.) of 3-hydroxy-2-(hydroxymethyl)-2-methylpropanoic acid (bisMPA), and 21.2 g of KOH (purity 90%, 338.9 mmol, 1.01 eq.) were suspended in 250 mL DMF and heated to 100°C. After 1 h a homogenous solution was formed and 48 mL of benzyl bromide was slowly added (69.1 g, 404.1 mmol, 1.2 eq.) using a dropping funnel and stirred at 100°C overnight. During the reaction a colorless solid precipitated. The reaction was cooled to room temperature and the solvent was removed by vacuum distillation. To the remaining liquid 250 mL of ethyl acetate and 250 mL *n*-hexane were added and extracted three times with 200 mL of water. The organic layer was dried over magnesium sulfate and the solvent was distilled off. To the crude product 75 ml of toluene was added, and 40.5 g of pure benzyl 3-hydroxy-2-(hydroxymethyl)-2-methylpropanoate crystallized overnight at -20°C (40.5 g, 54 %).

^{1}H NMR, Figure S3 (300 MHz, DMSO-d_6) δ 7.43 – 7.24 (m, 5H, 11, 12, 13, 14, 15), 5.09 (s, 2H, 16), 4.74 (t, J = 5.4 Hz, 2H, 1, 5), 3.56 (dd, J = 10.4, 5.5 Hz, 2H, 2', 4'), 3.46 (dd, J = 10.4, 5.5 Hz, 2H, 2'', 4''), 1.10 (s, 3H, 7).

^{13}C NMR, Figure S4 (75 MHz, DMSO-d_6) δ 174.69 (6), 136.68 (10), 128.42 (11, 15), 127.75 (13), 127.29 (12, 14), 65.14 (16), 64.04 (2), 50.43 (3), 16.99 (7).

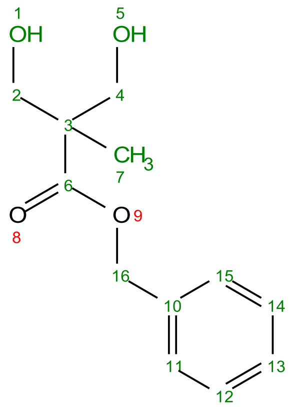

Figure S2: chemical structure of bisMPA-Bn with labelled atom positions.

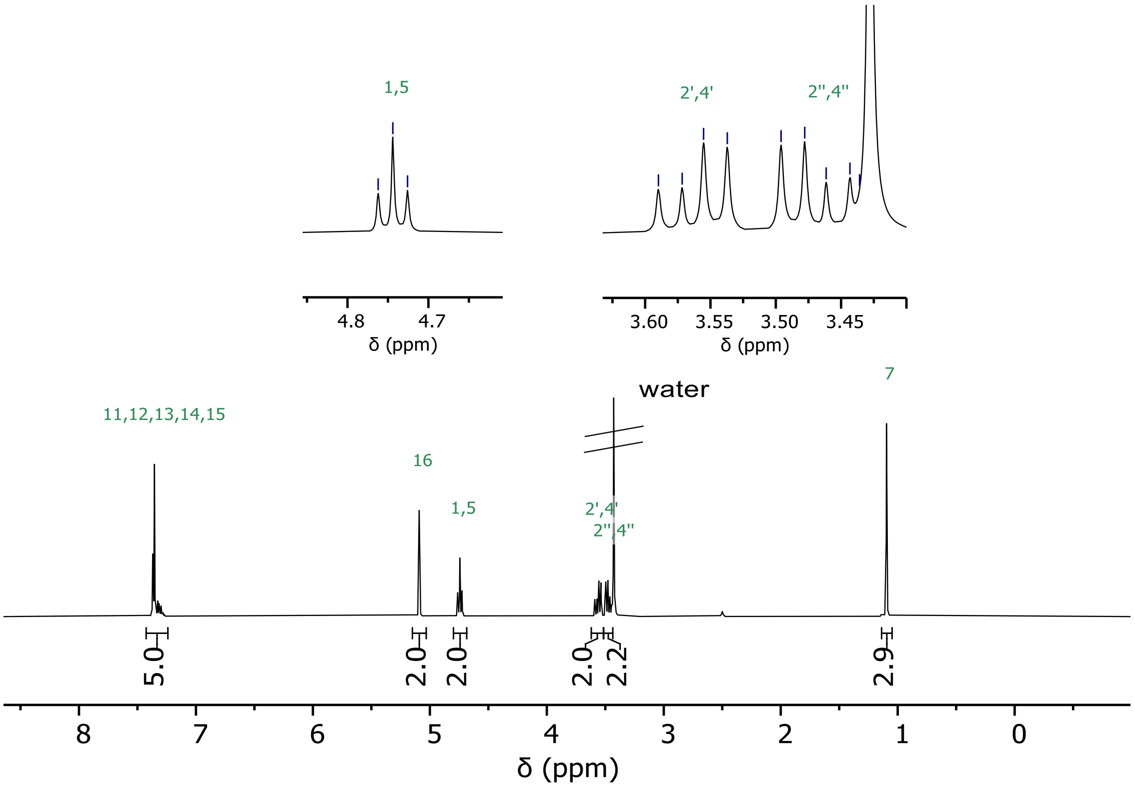

Figure S3: ^{1}H NMR spectrum (300 MHz, DMSO-d_6) of benzyl 3-hydroxy-2-(hydroxymethyl)-2-methylpropanoate

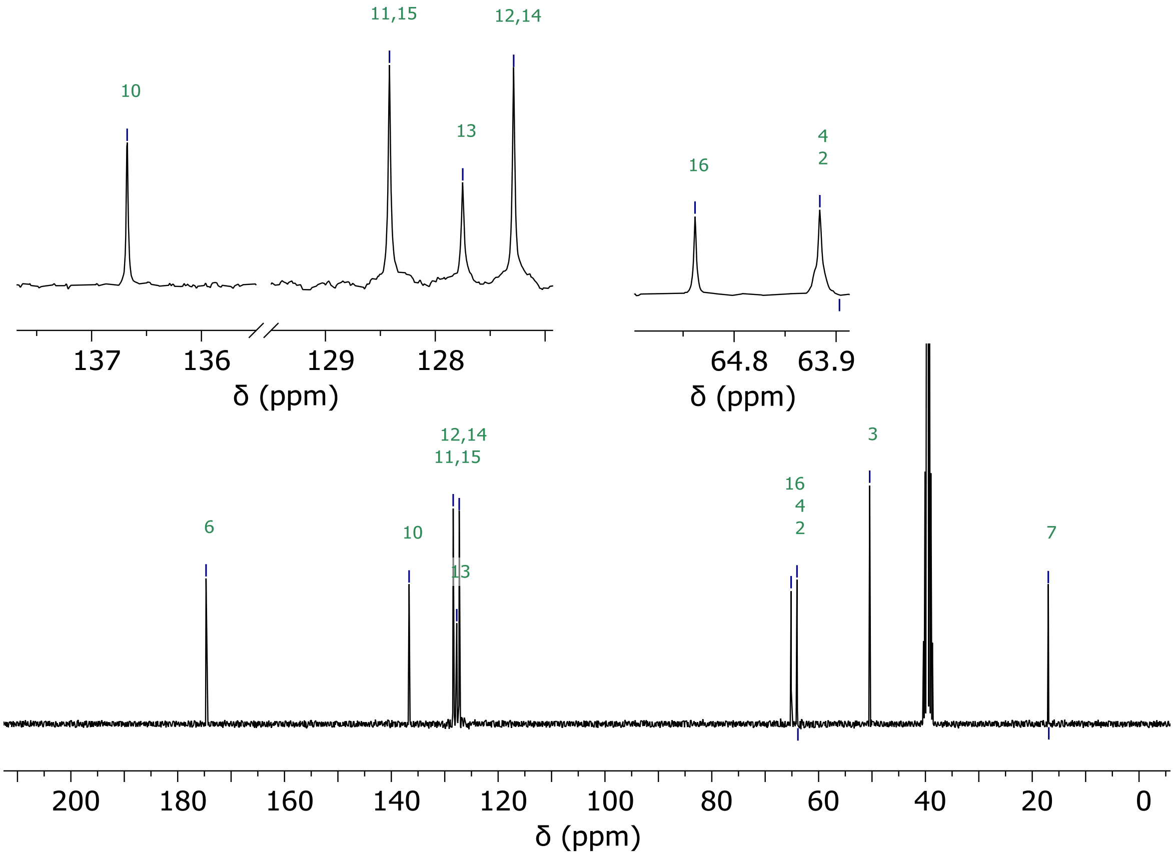

Figure S4: ^{13}C NMR spectrum (176 MHz, DMSO-d_6) of benzyl 3-hydroxy-2-(hydroxymethyl)-2-methylpropanoate

Synthesis of benzyl 5-methyl-2-oxo-1,3-dioxane-5-carboxylate (C)

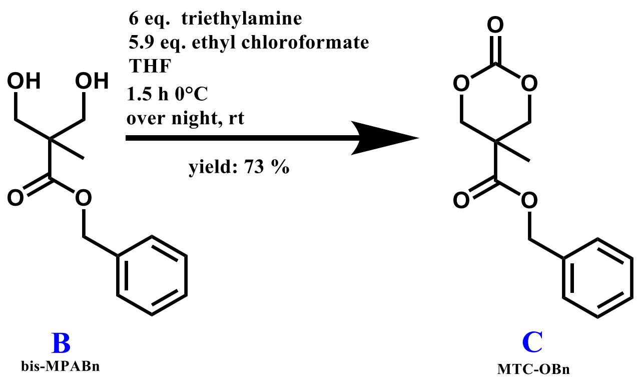

Figure S5: Synthesis of benzyl 5-methyl-2-oxo-1,3-dioxane-5-carboxylate abbreviated as MTC-OBn

Synthesis of benzyl 5-methyl-2-oxo-1,3-dioxane-5-carboxylate (MTC-OBn), Figure S5. Benzyl 5-methyl-2-oxo-1,3-dioxane-5-carboxylate was synthesized following an adapted literature procedure.[1–3] In a three-necked flask equipped with a mechanical stirrer and a dropping funnel, 23.46 g (105 mmol, 1.0 eq.) of bisMPA-Bn were dissolved in 1500 mL THF and cooled to 0°C using an ice bath. Under a nitrogen atmosphere 59 mL (620 mmol, 5.9 eq.) of ethyl chloroformate was then slowly added while stirring. Subsequently, 92 mL (665 mmol, 6.2 eq.) of triethylamine was added over a 1.5-hour period. The reaction mixture was allowed to warm to room temperature overnight, during which a white precipitate formed. The solution was then filtered, and concentrated under vacuum. To the remaining yellow oil, a solvent mixture of THF and Et_2O (1:1) was added, and colorless fine needles formed overnight (19.11 g, 73%).

^{1}H NMR, Figure S7 (300 MHz, $CDCl_3$) δ 7.46 – 7.27 (m, 5H), 5.22 (s, 2H), 4.70 (d, J = 10.8 Hz, 2H), 4.20 (d, J = 10.8 Hz, 2H), 1.33 (s, 3H).

^{13}C NMR, Figure S8 (75 MHz, $CDCl_3$) δ 171.04 (7), 147.52 (6), 134.89 (11), 128.92 (13, 15), 128.90 (14), 128.37 (12, 16), 73.08 (2, 4), 68.06 (10), 40.37 (3), 17.74 (18).

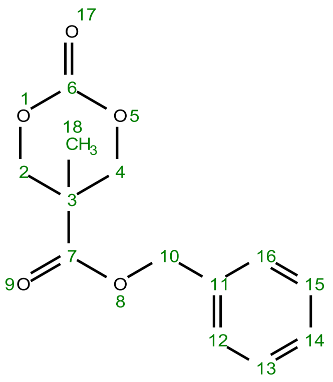

Figure S6: chemical structure of MTC-OBn with labelled atom positions.

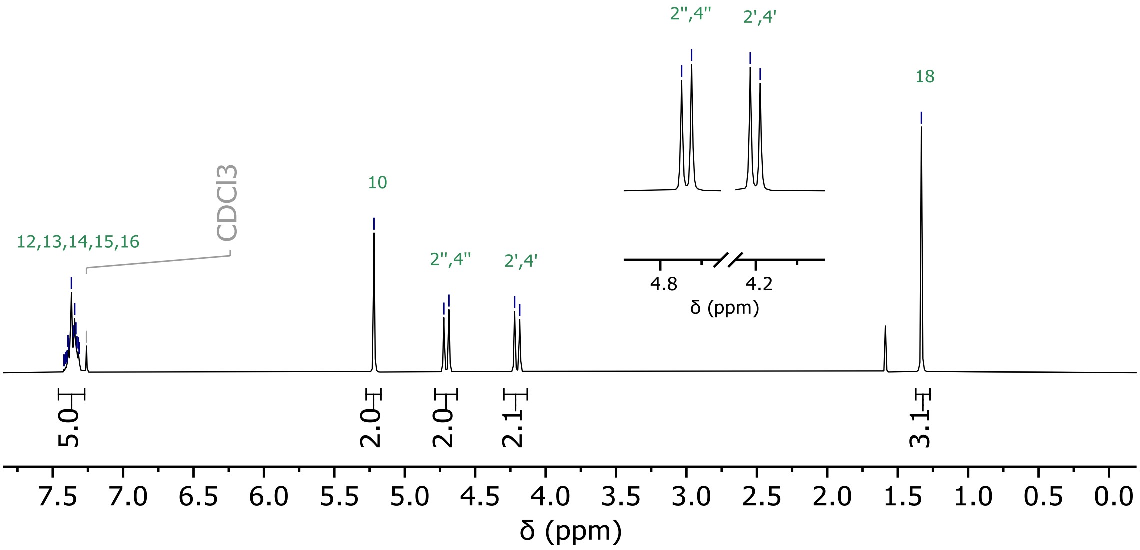

Figure S7: ^{1}H NMR spectrum (300 MHz, $CDCl_3$) of benzyl 5-methyl-2-oxo-1,3-dioxane-5-carboxylate

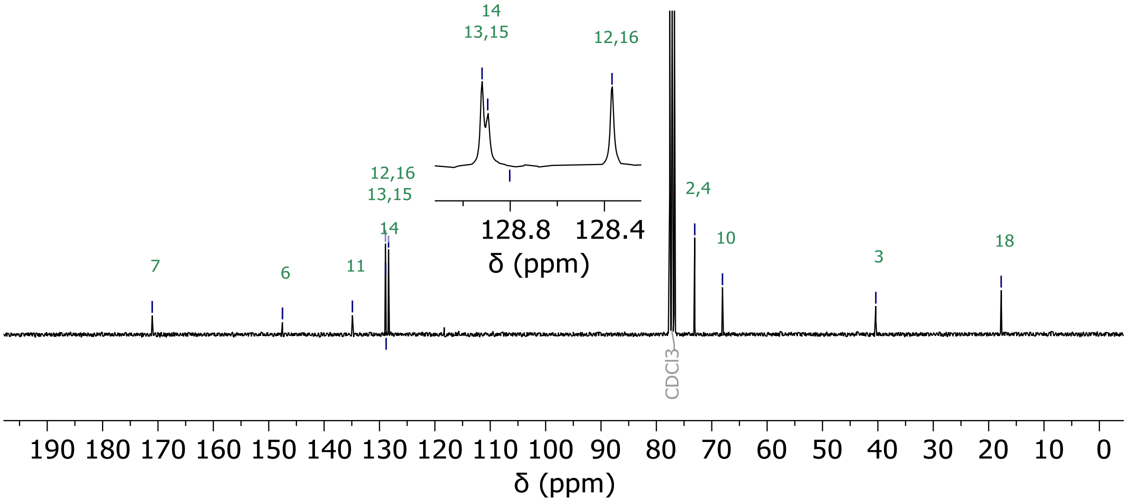

Figure S8: ^{13}C NMR spectrum (75 MHz, $CDCl_3$) of benzyl 5-methyl-2-oxo-1,3-dioxane-5-carboxylate

Synthesis of 5-methyl-2-oxo-1,3-dioxane-5-carboxylic acid (D)

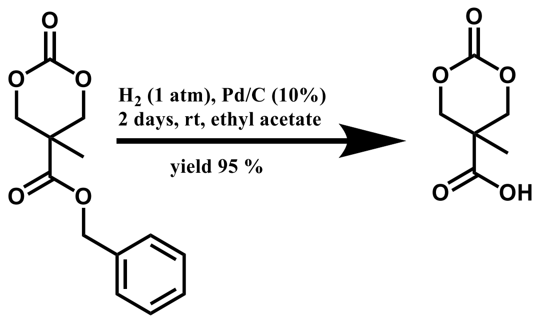

C **D**

Figure S9: Synthesis of 5-methyl-2-oxo-1,3-dioxane-5-carboxylic acid abbreviated as MTC-OH

Synthesis of 5-methyl-2-oxo-1,3-dioxane-5-carboxylic acid (MTC-OH), Figure S9. The synthesis was conducted following a previously reported literature procedure.[1–3] In a 500 mL round-bottom flask, 14.8 g (59 mmol, 1 eq.) of MTC-OBn were placed and dissolved in 150 mL of ethyl acetate. Then, 0.5 g of Pd/C (10%) was added, and the mixture was stirred under a hydrogen atmosphere. The progress of the reaction was monitored using TLC. After two days, complete conversion was achieved. The Pd/C catalyst was removed by filtration through Celite, and the filtrate was concentrated, yielding MTC-OH as colorless crystals (9.0 g, 95 %).

^{1}H NMR Figure S11, 250 MHz, DMSO-d_6) δ 13.37 (s, 1H, 10), 4.53 (d, *J* = 10.0 Hz, 2H, 2, 4), 4.30 (d, *J* = 10.0 Hz, 2H, 2, 4), 1.16 (s, 3H, 8).

^{13}C NMR, Figure S12 (75 MHz, DMSO-d_6) δ 173.34 (9), 147.36 (6), 72.69 (2, 4), 39.32 (3), 16.43 (8).

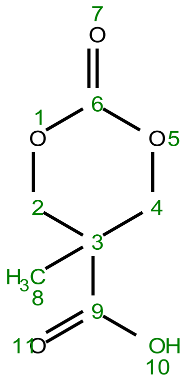

Figure S10: chemical structure of benzyl vinyl ether with labelled atom positions.

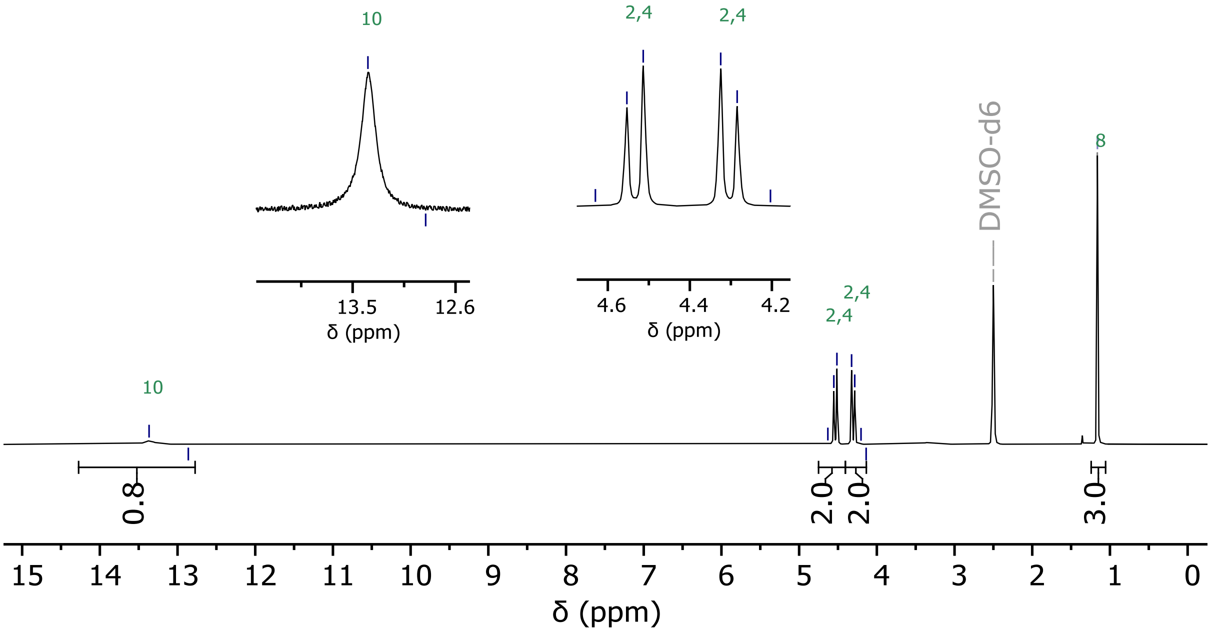

Figure S11: ^{1}H NMR spectrum (250 MHz, DMSO-d_6) of 5-methyl-2-oxo-1,3-dioxane-5-carboxylic acid

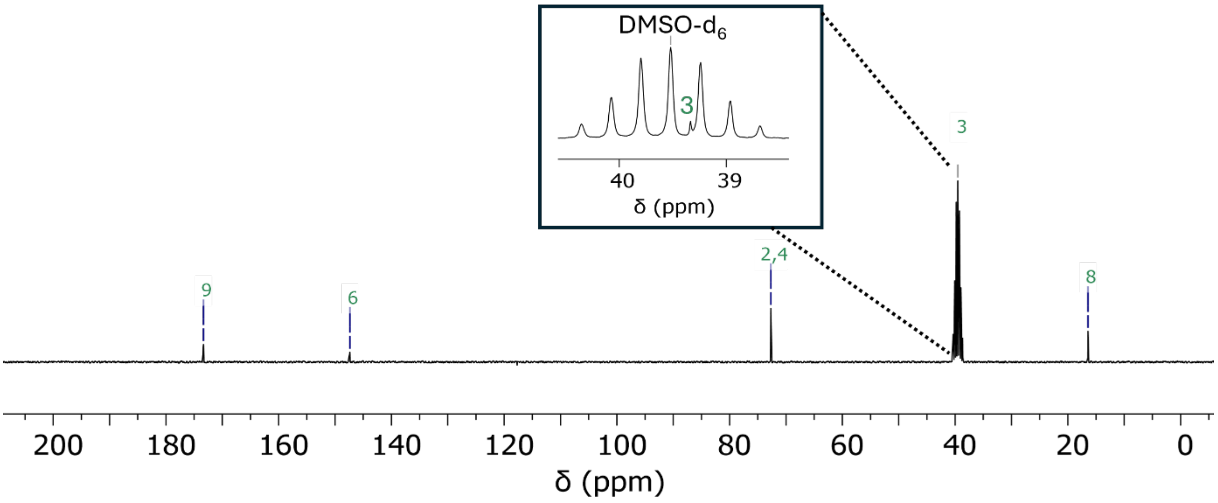

Figure S12: ^{13}C NMR spectrum (75.48 MHz, DMSO-d_6) of 5-methyl-2-oxo-1,3-dioxane-5-carboxylic acid

Synthesis of the pyridinium-P(styrene sulfonate) solid phase catalyst:

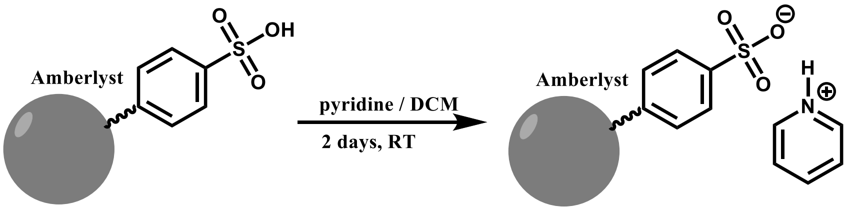

Figure S13: Schematic overview of the synthesis of pyridinium P(styrensulfoante)

Synthesis of the pyridinium-P(styrene sulfonate) solid phase catalyst, (Figure S13). Amberlyst^{R}15H, which is a commercially available crosslinked polystyrene sulfonic acid, was placed into a flask and submerged in DCM. The mixture was cooled in an ice bath to 0 °C, and pyridine was added gradually. The flask was gently rotated to stir the mixture for two days. The pyridinium-P(styrene sulfonate) was then separated by filtration, washed with DCM, and dried under 8 mbar vacuum for an additional two days. The catalyst was subsequently stored at room temperature.

Synthesis of 1-ethoxyethyl dodecanoate

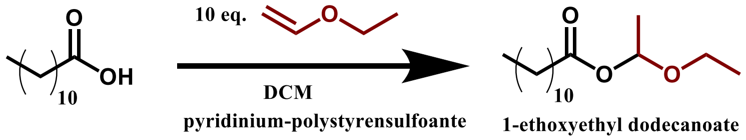

Figure S14: Synthesis of 1-ethoxyethyl dodecanoate.

Synthesis of 1-ethoxyethyl dodecanoate, (Figure S14). 0.5 g of 1-dodecanoate (2.5 mmol, 1 eq) was dissolved in 5 mL of DCM in a 20 mL reaction vial. To this solution 1.4 mL (3 eq. 7.8 mmol) of ethyl vinyl ether (previously filtered through a short column packed with basic aluminum oxide to remove polymeric impurities) was added. Subsequently, 0.5 g of the previously prepared pyridinium-P(styrene sulfonate) was added. The vial was sealed, and the mixture was stirred overnight at room temperature by rotating.

After completion of the reaction, the mixture was filtered through a PTFE-syringe filter (250 nm) to remove the solid catalyst. To the filtrate, 1 mL of *n*-heptane was added, and DCM and excess ethyl vinyl ether were removed by distillation under reduced pressure (down to 100 mbar at room temperature). The remaining solution in *n*-heptane was treated with calcium hydride and stirred for 5 hours. Volatile organic compounds, including *n*-heptane, were subsequently removed by overnight distillation under high vacuum (1×10^{-3} mbar) at room temperature. The resulting suspension was dissolved in DCM and filtered to remove any residual CaH_2. DCM was then removed by distillation, yielding 612 mg of 1-ethoxyethyl dodecanoate as a slightly yellow oil (yield: 90%).

^{1}H NMR, Figure S16, (300 MHz, CD_2Cl_2): δ 5.90 (q, *J* = 5.2 Hz, 1H, 13), 3.58 (m, 2H, 15), 2.29 (t, *J* = 7.5 Hz, 2H, 11), 1.59 (q, *J* = 7.1 Hz, 2H, 10), 1.34 (d, *J* = 5.2 Hz, 3H, 14), 1.27 (m, 16H, 2 , 3, 4, 5, 6, 7, 8), 1.17 (t, *J* = 7.1 Hz, 3H, 16), 0.88 (t, *J* = 6.9 Hz, 3H, 1).

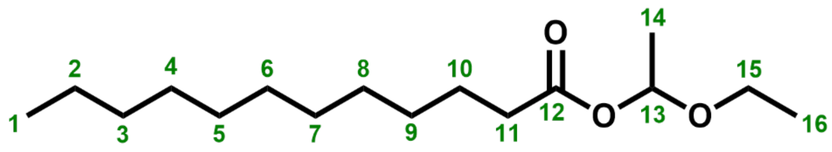

Figure S15: Chemical structure of 1-ethoxyethyl dodecanoate with labelled atom positions

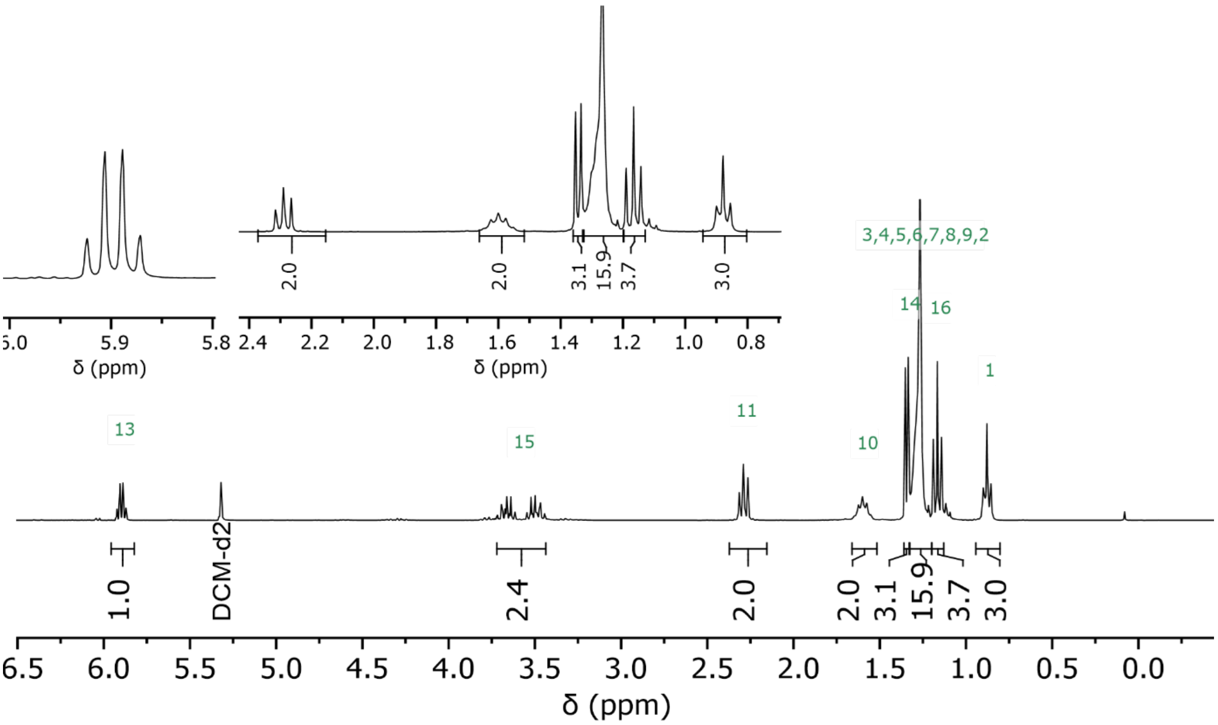

Figure S16: ^{1}H NMR spectrum (300 MHz, CD_2Cl_2) of 1-ethoxyethyl dodecanoate.

Synthesis of hemiacetal ester functionalized six-membered carbonate monomers.

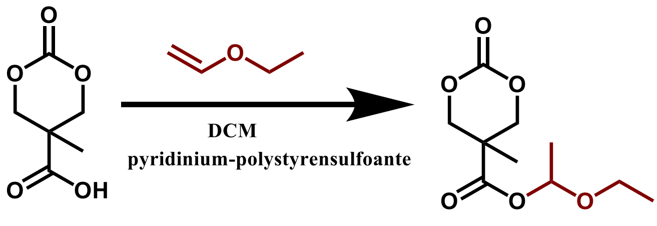

Figure S17: Synthesis of hemiacetal ester functionalized six-membered carbonate monomers

Synthesis of 1-ethoxyethyl 5-methyl-2-oxo-1,3-dioxane-5-carboxylate (Figure S17). MTC-OH (1.035 g, 1 eq) was suspended in 10 mL of DCM in a 20 mL reaction vial. To this suspension, 3.4 mL of ethyl vinyl ether (previously filtered through a short column packed with basic aluminum oxide to remove polymeric impurities) was added. Subsequently, 1 g of the previously prepared Pyridinium-P(styrene sulfonate) was added, and the mixture was stirred by rotating or shaking the reaction flask at room temperature for one day. The reaction was complete when all MTC-OH had been dissolved. Note that if a stir bar is used, the solid particles may get ground, making it difficult to assess the reaction progress based on the complete dissolution of MTC-OH. The catalyst is removed by filtration, followed by the addition of CaH_2. All volatile organic compounds are distilled off at room temperature under reduced pressure ($1\cdot10^{-3}$ mbar for at least one day). 1-ethoxyethyl 5-methyl-2-oxo-1,3-dioxane-5-carboxylate was obtained as a slightly yellow liquid (1.4533 g, 97%), which was pure as determined by NMR. The monomer was stored over CaH_2 at -20°C.

^{1}H NMR, Figure S19 (400 MHz, CD_2Cl_2) δ 6.00 (q, J = 5.2 Hz, 1H), 4.71 – 4.64 (m, 2H, 2'', 4''), 4.23 – 4.16 (m, 2H, 2', 4'), 3.68 (dq, J = 9.5, 7.1 Hz, 1H, 15''), 3.53 (dq, J = 9.5, 7.1 Hz, 1H, 15'), 1.41 (d, J = 5.2 Hz, 3H), 1.29 (s, 3H, 9), 1.18 (t, J = 7.1 Hz, 4H).

^{13}C NMR, Figure S21 (101 MHz, CD_2Cl_2) δ 171.22 (8), 98.72 (11), 73.16 (2, 4), 65.17 (15), 40.66 (3), 20.59 (14), 17.19 (9), 14.96 (16). For ^{1}H-^{1}H COSY see Figure S20, for ^{1}H-^{13}C HSQC see Figure S22, for ^{1}H-^{13}C HMBC see Figure S23.

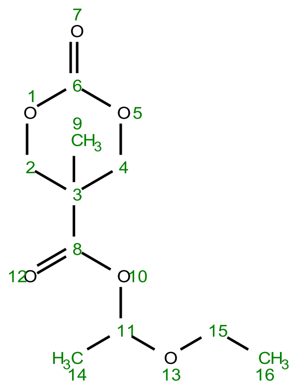

Figure S18: chemical structure of 1-ethoxyethyl 5-methyl-2-oxo-1,3-dioxane-5-carboxylate with labeled atom positions.

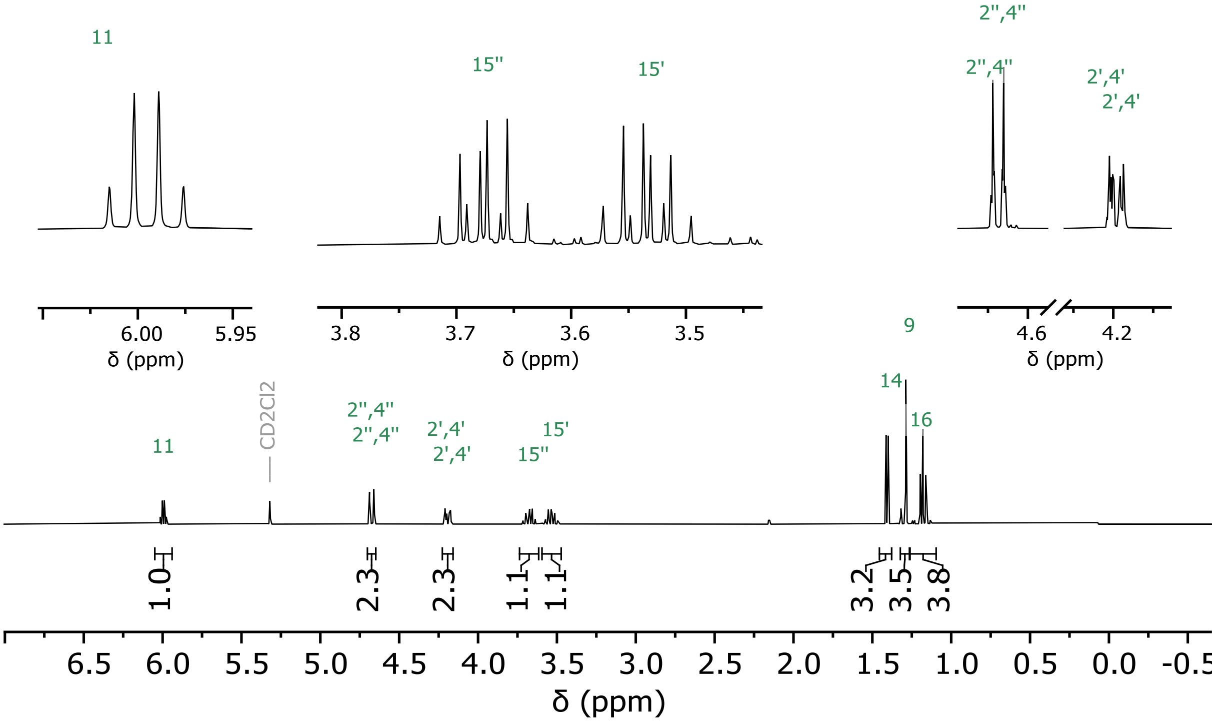

Figure S19: ^{1}H NMR spectrum (400 MHz, CD_2Cl_2) of 1-ethoxyethyl 5-methyl-2-oxo-1,3-dioxane-5-carboxylate.

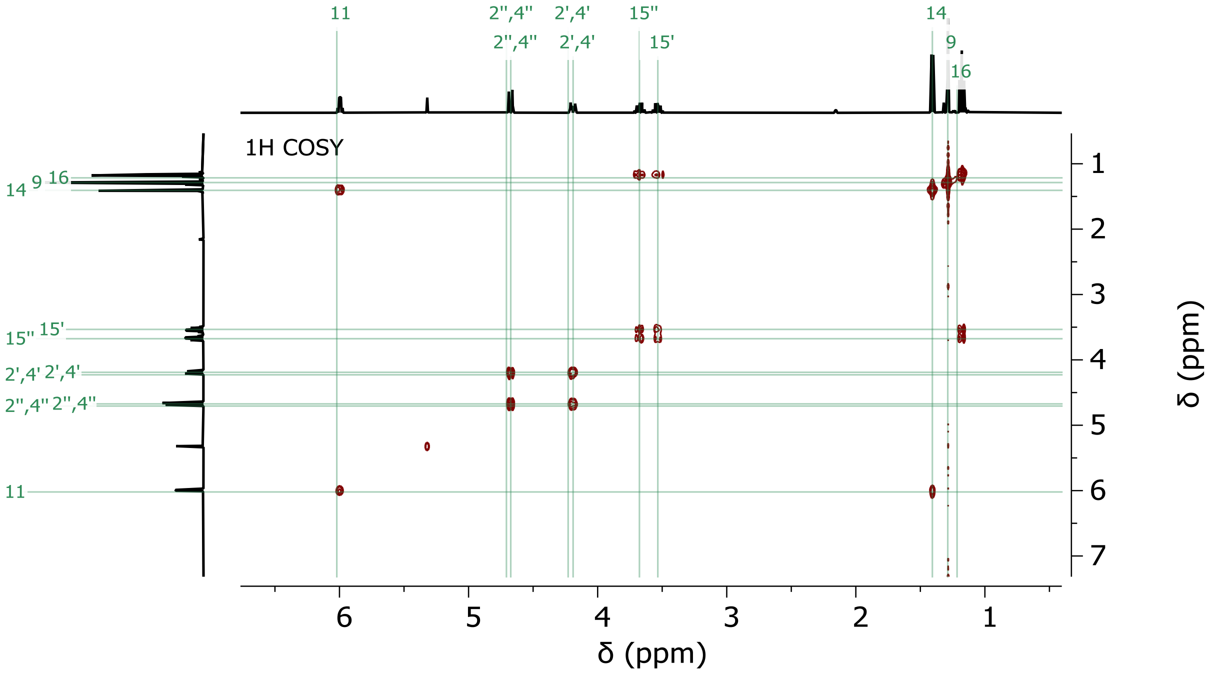

Figure S20: ^{1}H-^{1}H COSY NMR spectrum (400 MHz, CD_2Cl_2) of 1-ethoxyethyl 5-methyl-2-oxo-1,3-dioxane-5-carboxylate.

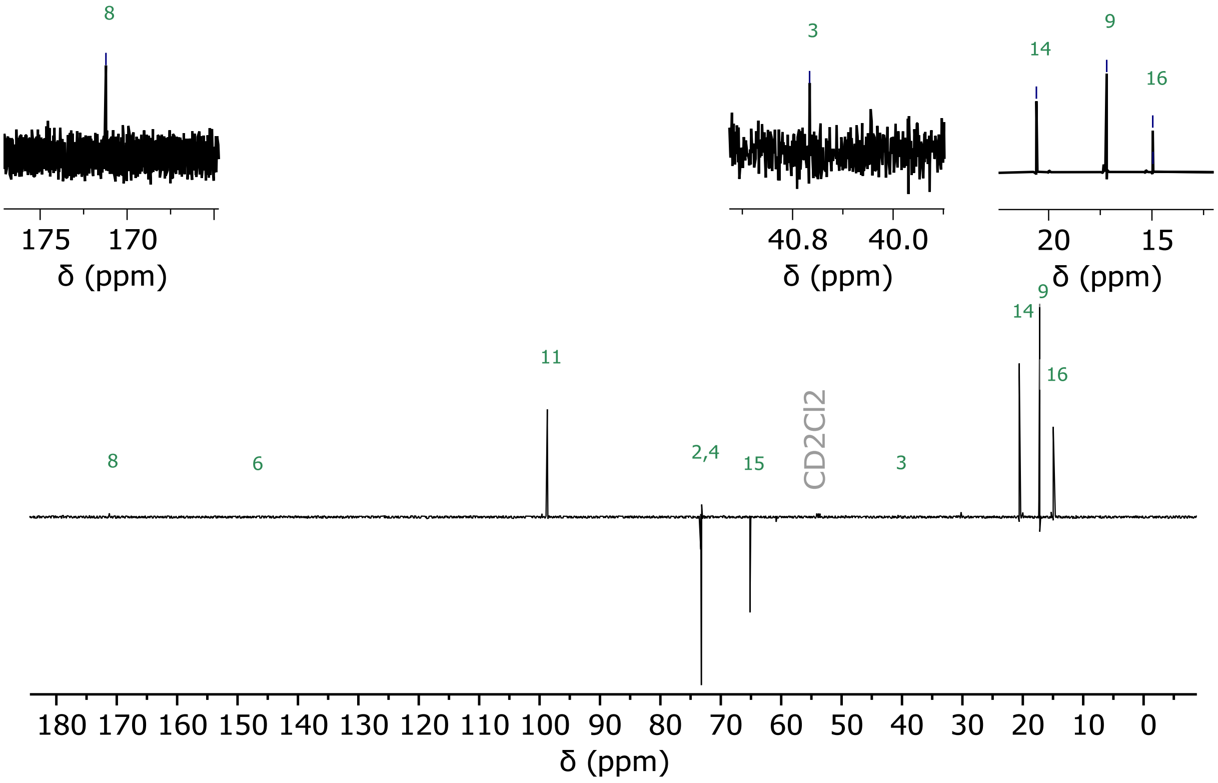

Figure S21: ^{13}C NMR spectrum (101 MHz, CD_2Cl_2) of 1-ethoxyethyl 5-methyl-2-oxo-1,3-dioxane-5-carboxylate.

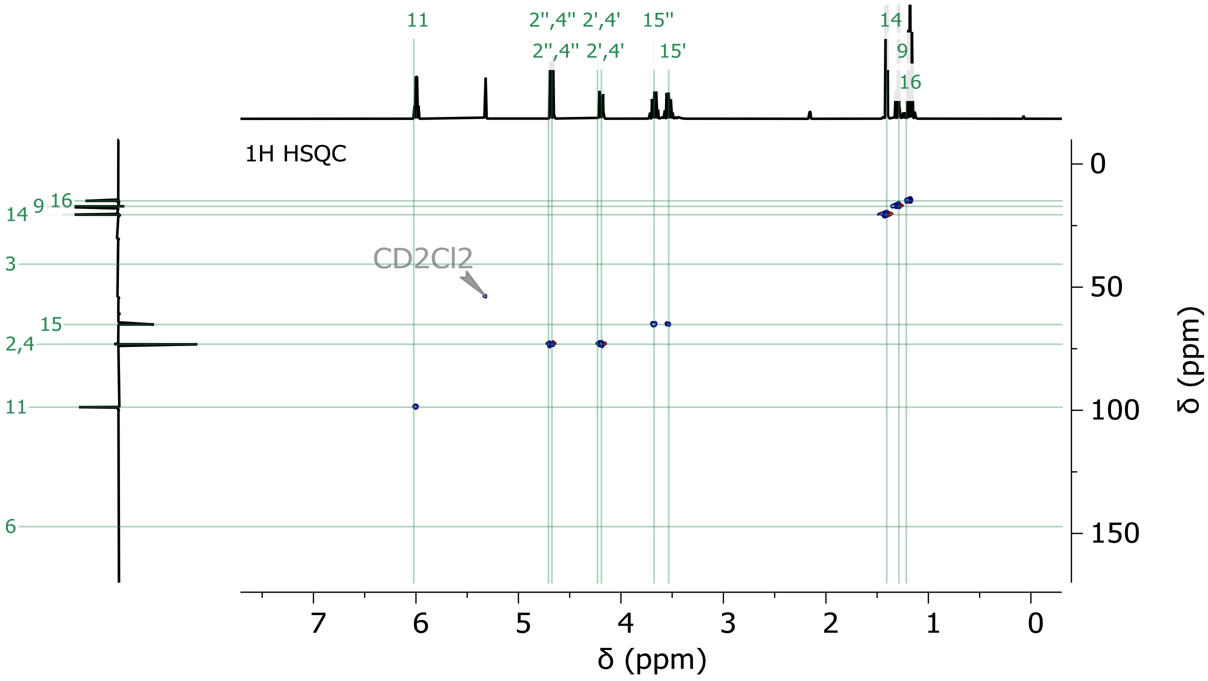

Figure S22: ^{1}H-^{13}C HSQC spectrum of 1-ethoxyethyl 5-methyl-2-oxo-1,3-dioxane-5-carboxylate.

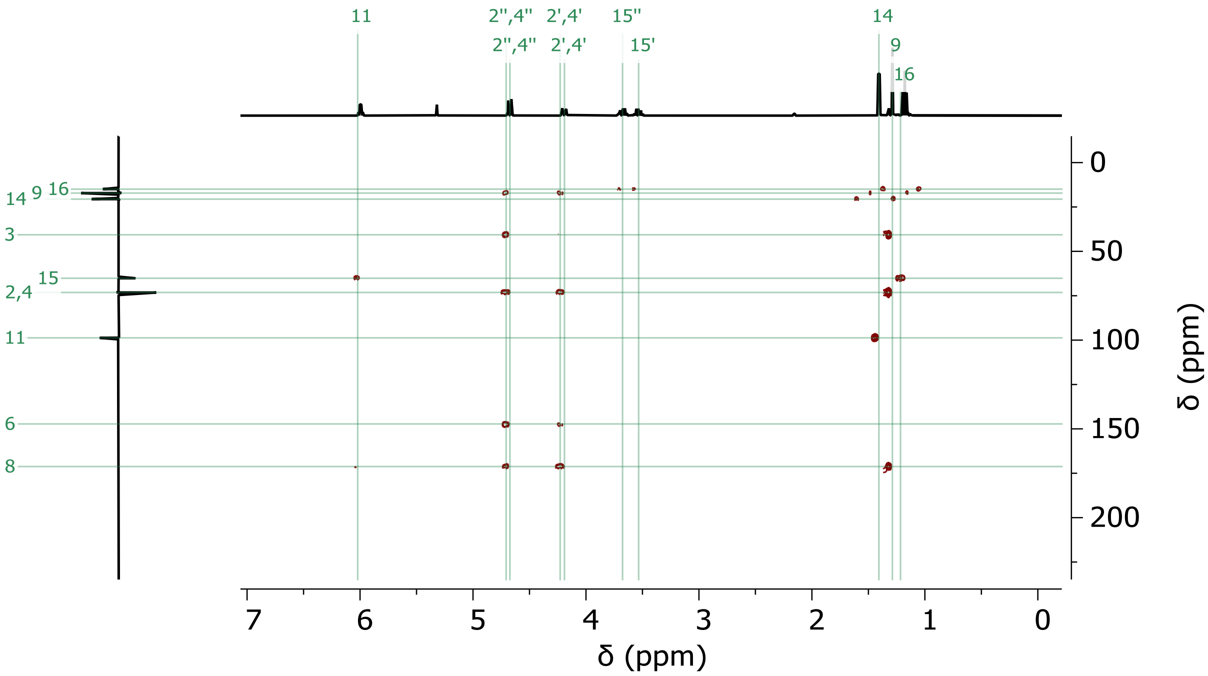

Figure S23: ^{1}H-^{13}C HMBC spectrum of 1-ethoxyethyl 5-methyl-2-oxo-1,3-dioxane-5-carboxylate.

Polymerizations

Kinetic analysis of the hemiacetal ester stability in presence of alcohols.

Kinetic analysis of the stability of 1-ethoxyethyl dodecanoate in presence of pyrene butanol dissolved in ACN-d_3 (Figure 3).

A 0.5 M stock solution of 1-ethoxyethyl dodecanoate in ACN-d_3 was prepared and stirred over CaH_2. Prior to use, the CaH_2 was removed by filtration, and a baseline ^1H NMR spectrum was recorded. Subsequently, 53 mg of pyrene butanol (0.201 mmol, 1.03 eq.) was added to 390 µL of the stock solution, and the reaction progress was monitored by sequentially recording^1H NMR spectra.

Kinetic analysis of the stability of 1-ethoxyethyl dodecanoate in presence of pyrene butanol (Figure 3).

A 0.5 M stock solution of 1-ethoxyethyl dodecanoate in CD_2Cl_2 was prepared and stirred over CaH_2. Prior to use, the CaH_2 was removed by filtration, and a baseline ^1H NMR spectrum was recorded. Subsequently, 62 mg of pyrene butanol (0.226 mmol, 1.15 eq.) was added to 390 µL of the stock solution, and the reaction progress was monitored by sequentially recording^1H NMR spectra.

Kinetic analysis of the stability of MTC-OEt-OEt in presence of bisMPA-Bn or pyrene butanol (Figure S24). A 0.5 M stock solution of MTC-OEt-OEt in CD_2Cl_2 was prepared and stirred over CaH_2. Just before use, the CaH_2 was removed by filtration and to 390 µL of this stock solution (0.195 mmol, 1 eq.), 20 µL of toluene were added as an internal standard, and a baseline ^1H NMR spectrum was recorded. Subsequently, 2.9 µL of DIPEA were added, and the reaction was monitored by sequentially recording^1H NMR spectra over 2 hours. After this initial phase, 21.9 mg of bisMPA-Bn (0.098 mmol, 0.5 eq., equivalent to 1.0 eq. with respect to the OH functionality) was further added, and the reaction progress was further monitored by additional ^1H NMR measurements.

The kinetic analysis of MTC-OEt-OEt stability in the presence of pyrene butanol was conducted using a similar protocol. However, the DIPEA incubation period was reduced to 1 hour, as the stability under basic conditions had already been established by the previous experiment. Additionally, the amount of pyrene butanol was increased to 1 eq. (53.4 mg, 0.195 mmol) to achieve a 1:1 monomer-to-OH functionality ratio.

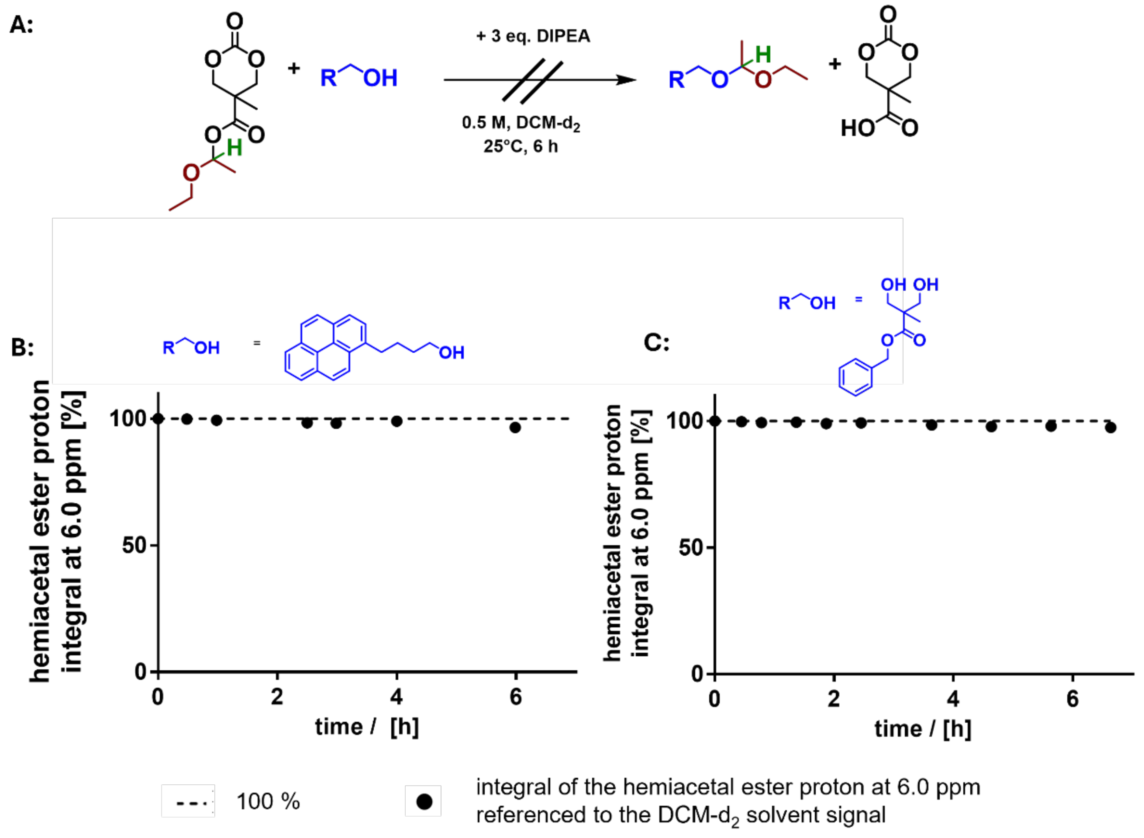

Figure S24: Kinetic analysis of the stability of MTC-OEt-OEt in presence of pyrene butanol (B) and bisMPA-Bn (C).

Homopolymerization

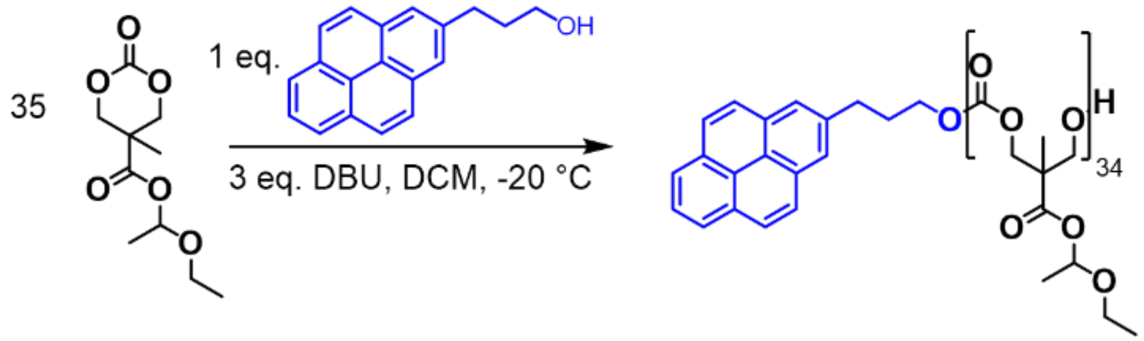

Figure S25: Reaction scheme of the homopolymerization of pyrene butanol-P(MTC-OEt-OEt)$_{33}$.

Homopolymerization of pyrene butanol-P(MTC-OEt-OEt)$_{33}$

The homopolymerization conditions were adapted from literature protocols for the polymerization of other six-membered carbonate monomers and modified.[4–7] A stock solution of monomer in dichloromethane was prepared at 1.4 M concentration and left to stir for a minimum of 3 days over calcium hydride. In a glovebox, 10.2 mg (0.037 mmol; 1 eq.) of pyrene butanol was weighed into a flame-dried Schlenk tube. Subsequently, the previously prepared monomer stock solution was filtered through a PTFE-syringe filter to remove CaH_2 and 928.5 µL (1.3 mmol; 35 eq.) was carefully added to ensure no undissolved pyrene butanol adhered to the glass tube wall and diluted to 0.5 M final monomer concentration with ~ 1.7 mL of DCM. The mixture was removed from the glovebox and then cooled under an inert atmosphere for at least 1 hour to -20°C using a cryostat. To this solution, 17 µL of DBU (111 µmol, 3 eq.), dissolved in a small amount of dry DCM, was added to initiate the polymerization. After 6 hours (at a conversion of approximately ~ 95%, as determined by NMR), the polymerization was quenched by precipitation in *n*-heptane at room temperature, followed by centrifugation at 9000 rpm for 15 minutes. The polymer pellet was then redissolved in DCM, and the precipitation purification procedure was repeated twice. Finally, the polymer was freeze dried with 2 mL of benzene for 24 hours at 1 x 10^{-3} mbar.

To follow the evolution of molecular weight with respect to monomer conversion, samples were taken at specific time points during the polymerization using a syringe. These samples were quenched by dilution in $CDCl_3$ and then analyzed using ^{1}H NMR spectroscopy. Subsequently, the chloroform solutions were analyzed by THF SEC (results are listed in Table S1 and plotted in Figure 4D).

Table S1: Evolution of molecular weight with respect to monomer conversion determined by ^{1}H NMR spectroscopy and THF SEC analysis (the molecular weight that was derived from the PMMA calibration at 254 nm was used for the UV detection at 344 nm).

**Conversion determined by ^{1}H NMR**	M_n^{prod} ($UV_{344\ nm}$-SEC)	PDI ($UV_{344\ nm}$-SEC)	M_n^{prod} (RI-SEC)	PDI (RI-SEC)
29	1326	1.6	1534	1.5
60	3260	1.4	2984	1.4
87	4721	1.1	5543	1.1
94	4976	1.09	5856	1.09

NMR characterization of pyrene butanol-P(MTC-OEt-OEt)$_{33}$

^{1}H NMR, Figure S26, (300 MHz, CD_2Cl_2) δ 8.24 – 7.78 (m, 9H), 5.86 (q, J = 5.2 Hz, 33H), 4.18 (s, 133H), 3.58 (dq, J = 9.3, 7.1 Hz, 39H), 3.42 (dq, J = 9.5, 7.1 Hz, 39H), 1.32 – 1.23 (m, 101H), 1.17 (s, 102H), 1.09 (t, J = 7.1 Hz, 109H). For ^{1}H-^{1}H COSY see Figure S27, for ^{1}H-^{13}C HSQC see Figure S28.

SEC $M_{n\ (RI)}$ ~6 500 g/mol, $Đ_{RI}$ = 1.11 (Figure S29).

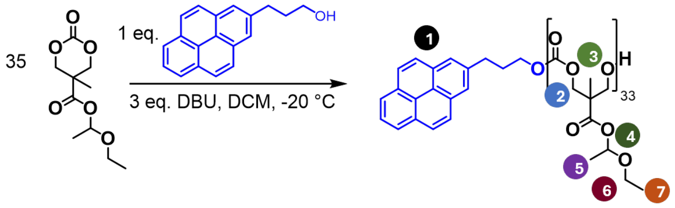

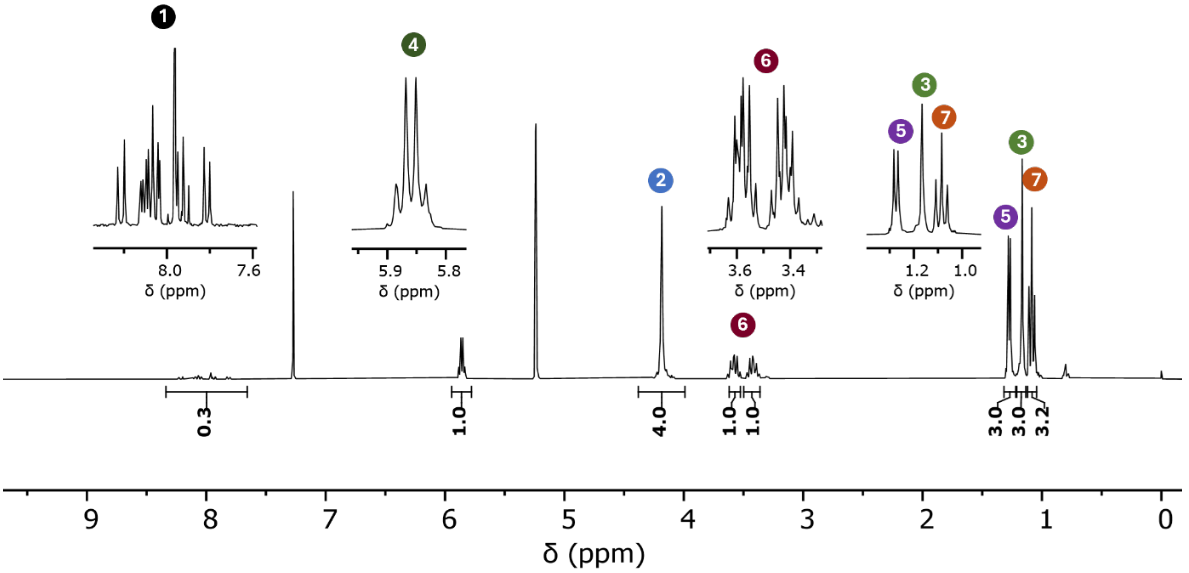

Figure S26: ^{1}H NMR spectrum (300 MHz, CD_2Cl_2) of pyrene butanol-P(MTC-OEt-OEt)$_{33}$ (integrals referenced to one monomer subunit).

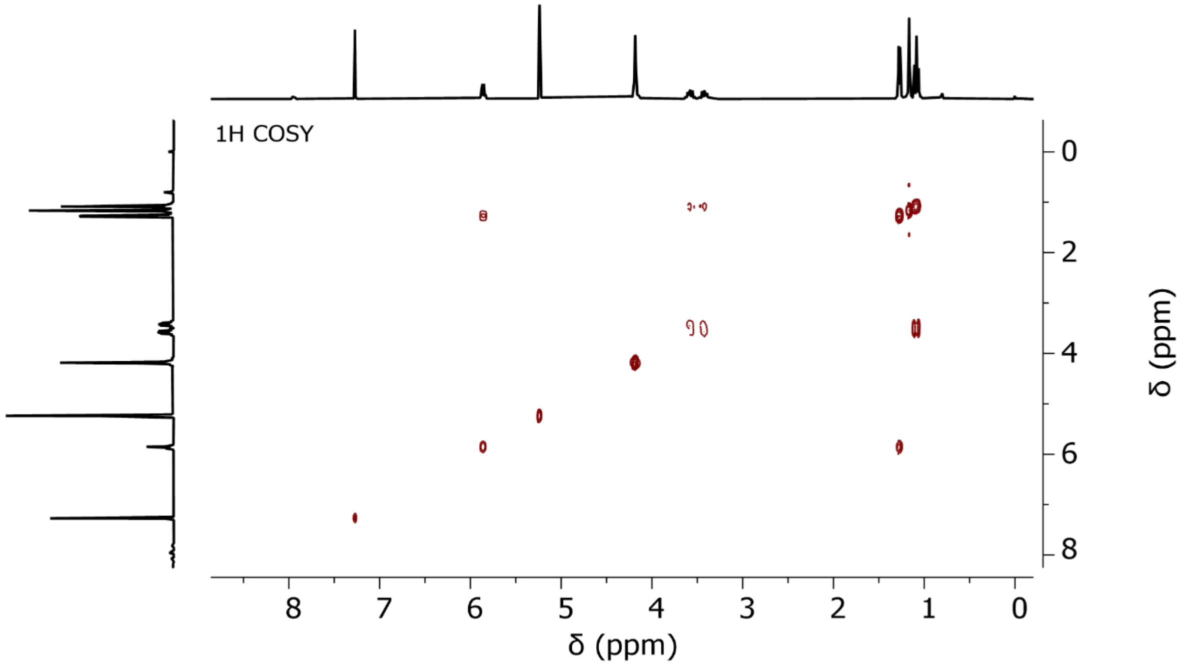

Figure S27: ^{1}H-^{1}H COSY NMR spectrum (300 MHz, CD_2Cl_2) of pyrene butanol-P(MTC-OEt-OEt)$_{33}$

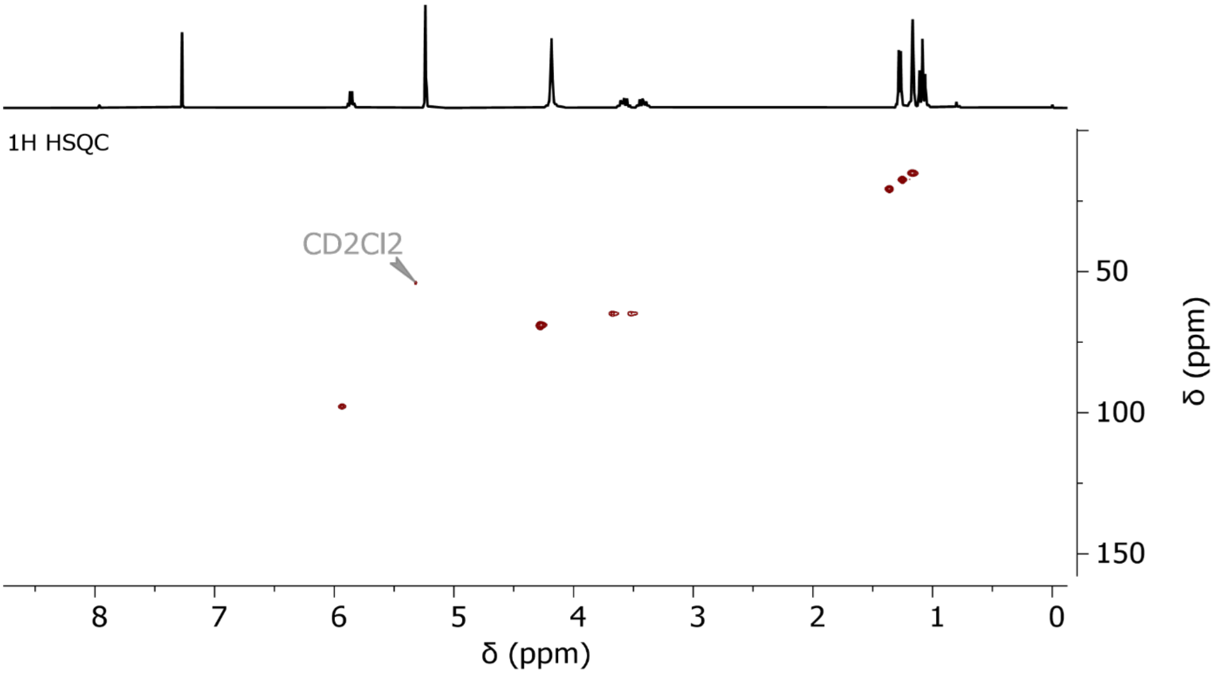

Figure S28: ^{1}H-^{13}C HSQC NMR spectrum (300 MHz, CD_2Cl_2) of pyrene butanol-P(MTC-OEt-OEt)$_{33}$

SEC trace analysis of pyrene butanol-P(MTC-OEt-OEt)$_{33}$

Table S2: Summary of the characterization of pyrene butanol-P(MTC-OEt-OEt)$_{33}$.

χ_n^{targ}	conv. [%]	$\chi_n^{product}$ (NMR)	M_n^{prod} (NMR)	M_n^{prod} (RI-SEC)	M_n^{prod} (UV-SEC)	$Đ_{UV\text{-}344\ nm}$	$Đ_{RI}$
35	≥ 95	~ 33	~ 7 900	~ 6 500	5 900	1.06	1.11

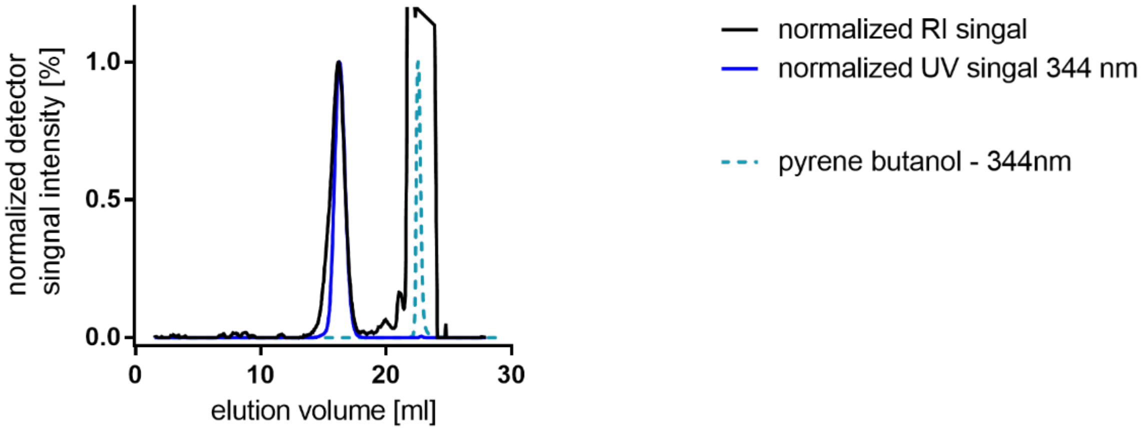

Figure S29: SEC trace analysis of pyrene butanol-P(MTC-OEt-OEt)$_{33}$ compared to the pyrene butanol itself.

Block Copolymerization

Block Copolymerization of $mPEG_{115}$-*b*-P(MTC-OEt-OEt)$_{11}$

The block copolymerization conditions were adapted from literature.[5,6,8] A stock solution of the monomer in dichloromethane was prepared at a concentration of 0.5 M and left to stir for ~3 days over calcium hydride. In a glove box, 100 mg of $mPEG_{5k}$-OH (20 µmol; 1 eq.) was weighed into a flame-dried Schlenk tube. Subsequently, the solution was filtered through a PTFE-syringe filter and 600 µL (300 µmol, 15 eq.) were carefully added to ensure no $mPEG_{5k}$-OH adhered to the glass tube wall. The mixture was then cooled for at least 1 hour to -20°C using a cryostat. To this solution, 3 µL of DBU (20 µmol; 1 eq.), dissolved in a small amount of dry DCM, were added to initiate the polymerization. After ~19 h and a conversion of ~85% (determined by NMR), the polymerization was quenched by precipitation in *n*-heptane at room temperature followed by centrifugation (9000 rpm, 15 min, rt). The polymer pellet was redissolved in 2 mL of DCM and precipitated for additional three times in -20°C cold diethyl ether. Finally, the polymer was freeze dried with 1 mL of benzene for 24 hours at 1 x 10^{-3} mbar.

^{1}H NMR, Figure S31, δ 5.95 (q, J = 5.4 Hz, 11H, 5), 4.27 (s, 45H, 3), 3.64 (s, 475H, 2), 3.52 (q, J = 7.4 Hz, 24H, 7), 3.38 – 3.37 (s, 3H, 1), 1.37 (d, J = 5.3 Hz, 34H, 6), 1.25 (s, 35H, 4), 1.19 (t, J = 7.1 Hz, 35H, 8).

SEC (THF) $M_{n\ (RI)}$ ~7 500 g/mol, $Đ_{RI}$ = 1.05 (Figure S32).

mPEG$_{115}$-OH

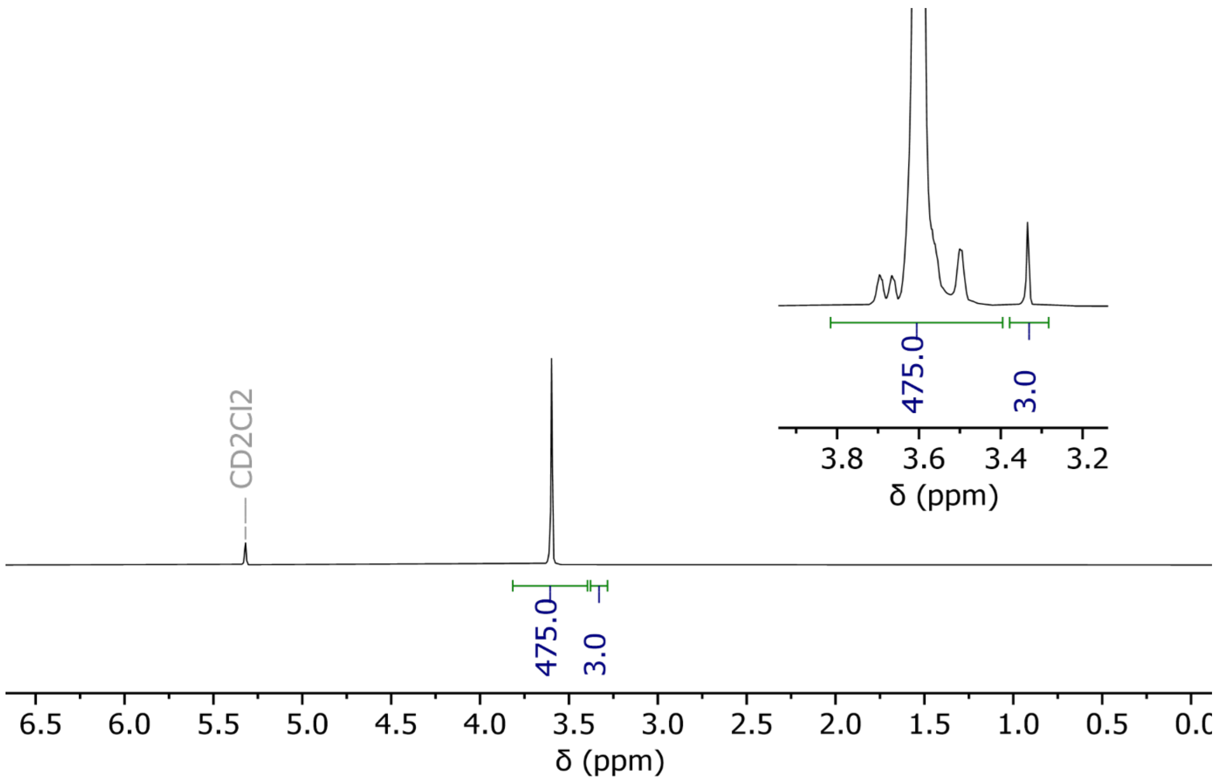

Figure S30: ^{1}H NMR spectrum (700 MHz, CD_2Cl_2) of mPEG$_{115}$-OH

mPEG$_{115}$-*b*-P(MTC-OEt-OEt)$_{11}$

mPEG$_{115}$-*b*-P(MTC-OEt-OEt)$_{11}$:

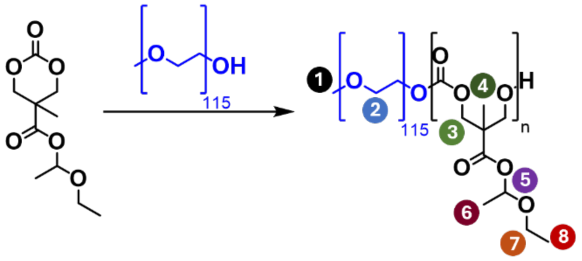

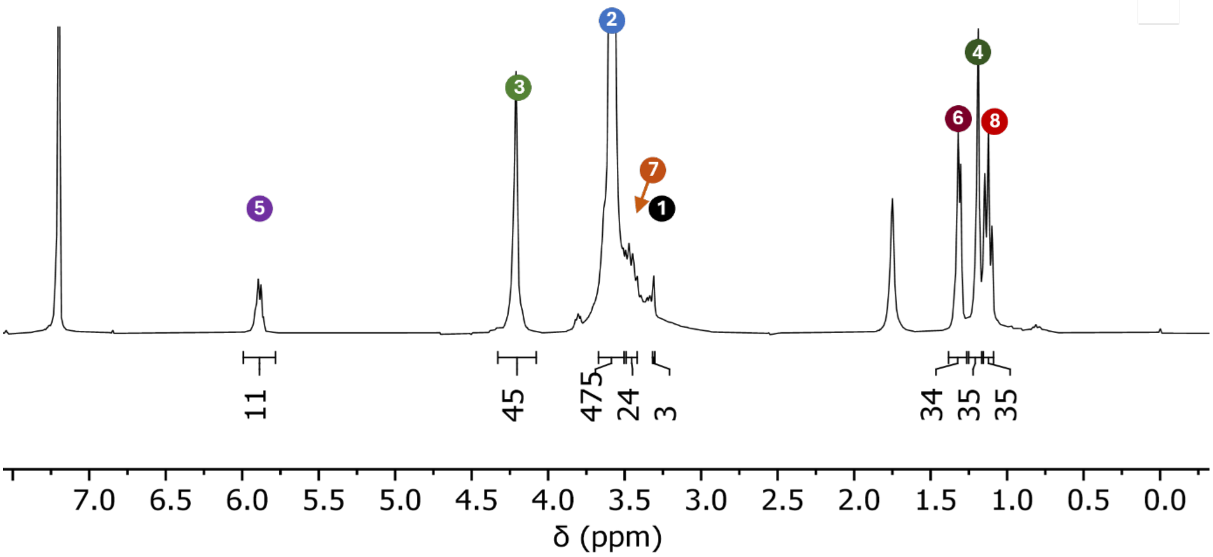

Figure S31: ^{1}H NMR spectrum (300 MHz, $CDCl_3$) of mPEG$_{115}$-*b*-P(MTC-OEt-OEt)$_{11}$

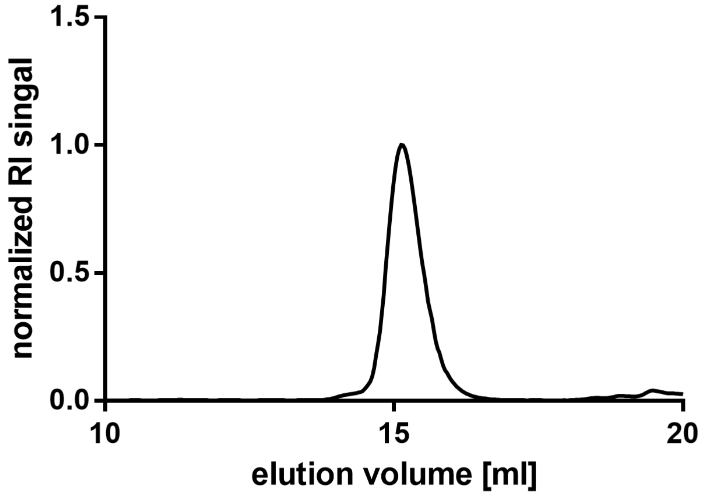

Figure S32: THF SEC RI-trace analysis of mPEG$_{115}$-*b*-P(MTC-OEt-OEt)$_{11}$

Hemiacetal ester deprotection

Deprotection by ethanol:

Deprotection by ethanol (Figure S34). 6 mg of pyrene butanol-P(MTC-OEt-OEt)$_{33}$ was dissolved in 450 µL of ethanol-d$_6$ by ultrasonication for approximately 2 minutes at room temperature. The solution was then directly transferred into an NMR tube and ^{1}H NMR spectra were measured sequentially over 60 hours. After the measurements, all volatile compounds were removed under vacuum, leaving 4 mg of the pure deprotected polymer as a white solid. The obtained polymer was characterized by ^{1}H NMR spectroscopy (Figure S33 and HFIP SEC Figure 6F).

^{1}H NMR, Figure S33, (400 MHz, DMSO-d$_6$) δ 8.41 – 7.90 (m, 9H, 1), 4.18 (s, 131H, 2), 1.32 – 1.01 (m, 104H, 3).

SEC (HFIP) $M_{n\,(RI)}$ ~4 100 g/mol, $Đ_{RI}$ = 1.13 (Figure 6B).

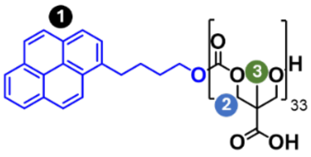

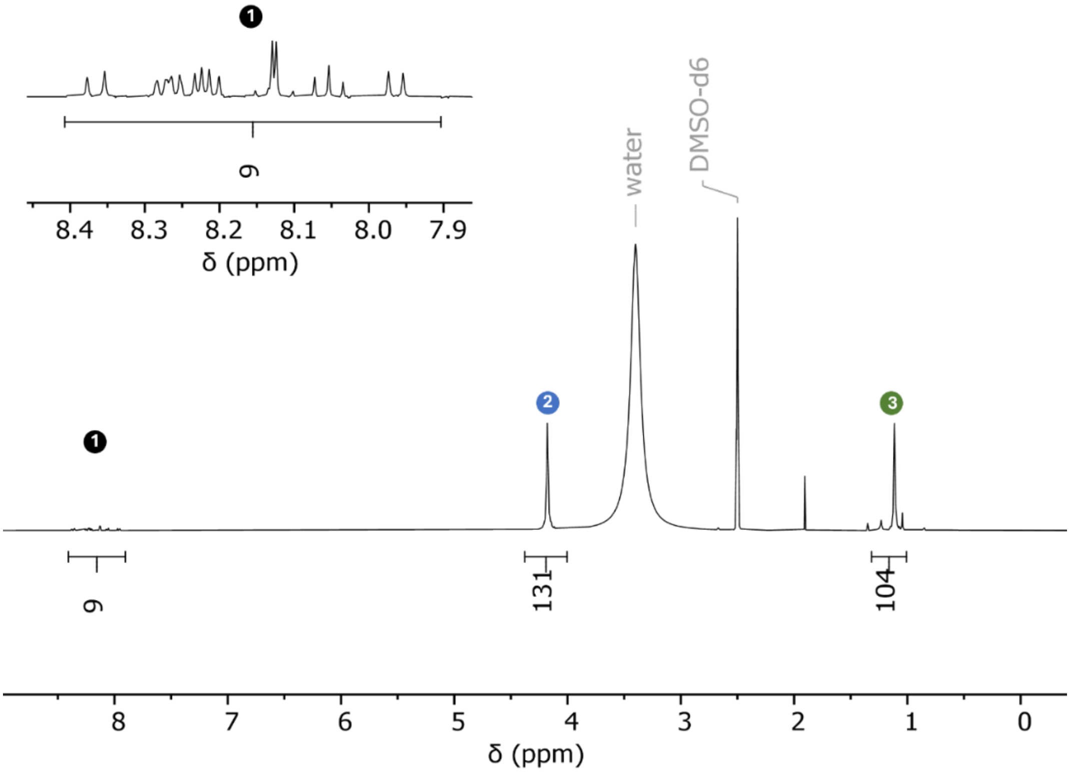

Figure S33: ^{1}H NMR spectrum (400 MHz, DMSO-d$_6$) of pyrene butanol-P(MTC-OH)$_{33}$ obtained by deprotection with ethanol. To exclude that differences in polymer relaxation times influence the determined degree of polymerization, we further varied the d1 time between 0.2 sec and 5 sec did not observe relevant differences.

Table S3: Summary of the polymer characterization obtained through deprotection with ethanol.

$\chi_n^{product}$ (NMR)	M_n^{prod} (NMR)	M_n^{prod} (RI-SEC) In HFIP	M_n^{prod} (UV_{344nm}-SEC) In HFIP	$Đ_{UV\text{-}344\ nm}$	$Đ_{RI}$
33	8 000	4 100	3 800	1.10	1.13

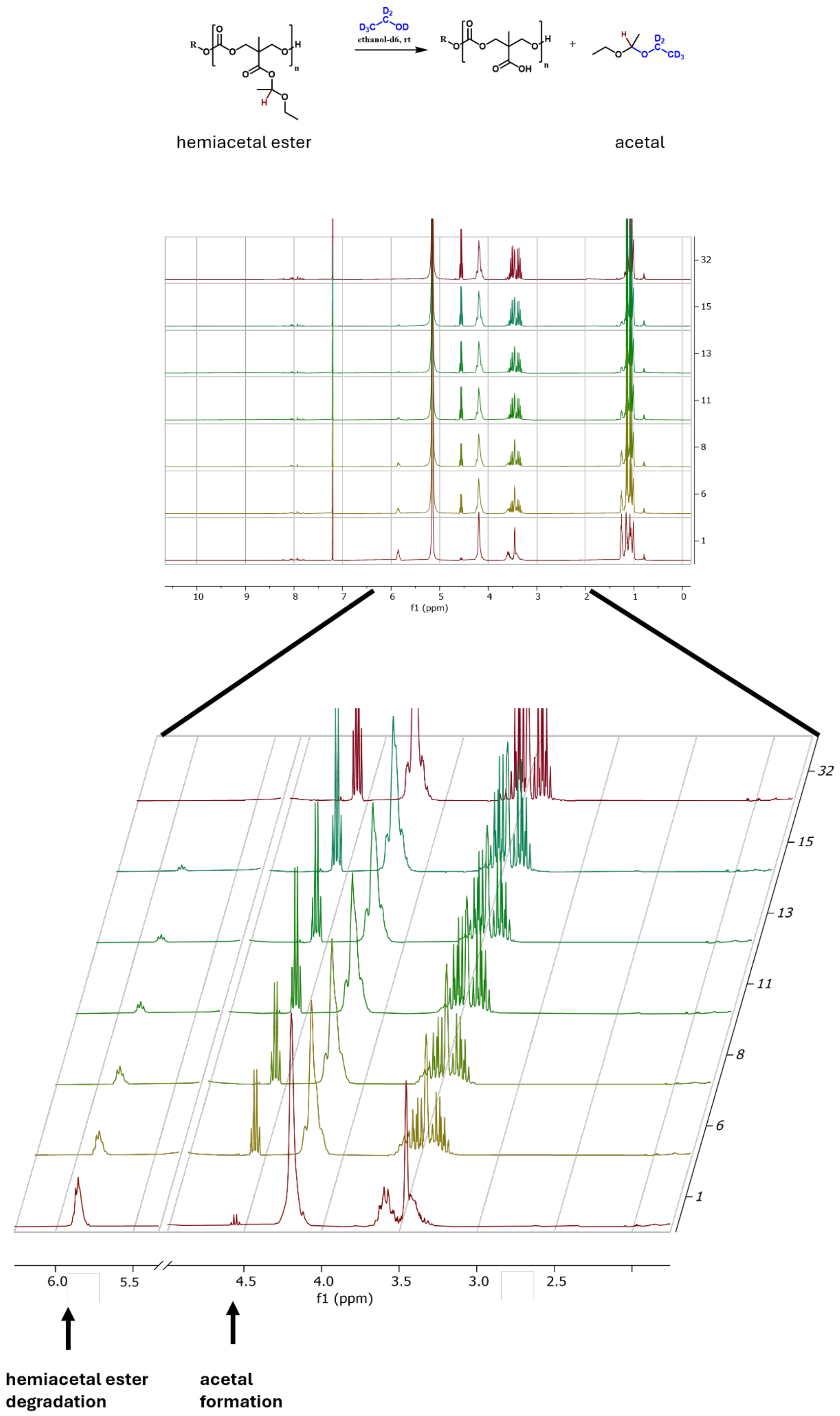

Figure S34: Hemiacetal ester side chain deprotection by ethanol-d_6 at room temperature.

Hemiacetal ester deprotection by thermolysis:

Thermogravimetric analysis. Pyrene butanol-P(MTC-OEt-OEt)$_{33}$ (10 - 20 mg) or pyrene butanol-P(MTC-OH)$_{33}$ (obtained by deprotection with ethanol) was placed in aluminum oxide crucibles (Netzsch) and heated at 140°C for 120 min under ambient air (25-100°C at 10 K/min, 100 -140°C 2K/min), with the mass loss being recorded. Afterward, the remaining polymer was analyzed by ^{1}H NMR spectroscopy (Figure S39) and SEC analysis (Figure S38).

Thermogravimetric analysis:

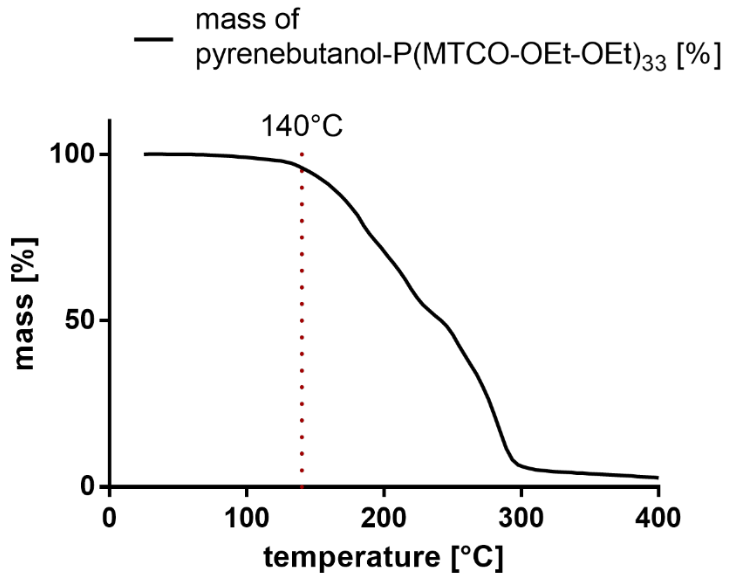

Figure S35: Thermogravimetric analysis of pyrene butanol-P(MTC-OEt-OEt)$_{33}$ (10 K/min).

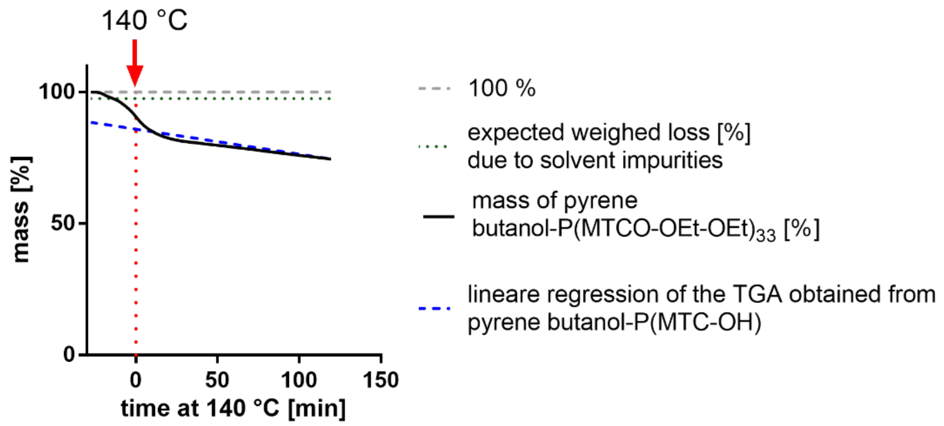

Figure S36: Thermogravimetric analysis of pyrene butanol-P(MTC-OEt-OEt)$_{33}$ at 140°C for 120 min. (25-100°C at 10 K/min, 100 -140°C 2K/min)

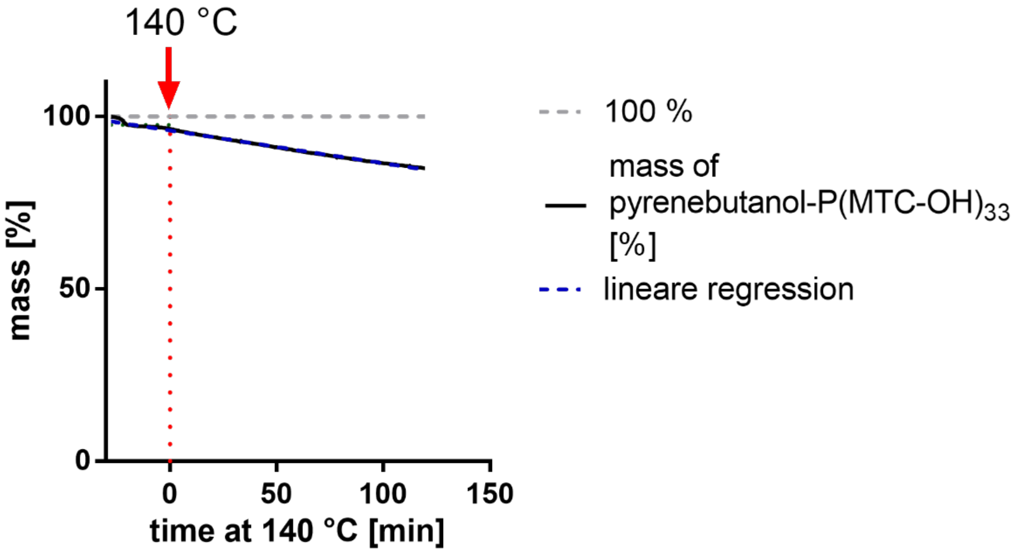

Figure S37: Thermogravimetric analysis of pyrene butanol-P(MTC-OH)$_{33}$ at 140°C for 120 min. (25-100°C at 10 K/min, 100 -140°C 2K/min). The deprotected polymer was obtained by deprotection with ethanol.

SEC analysis:

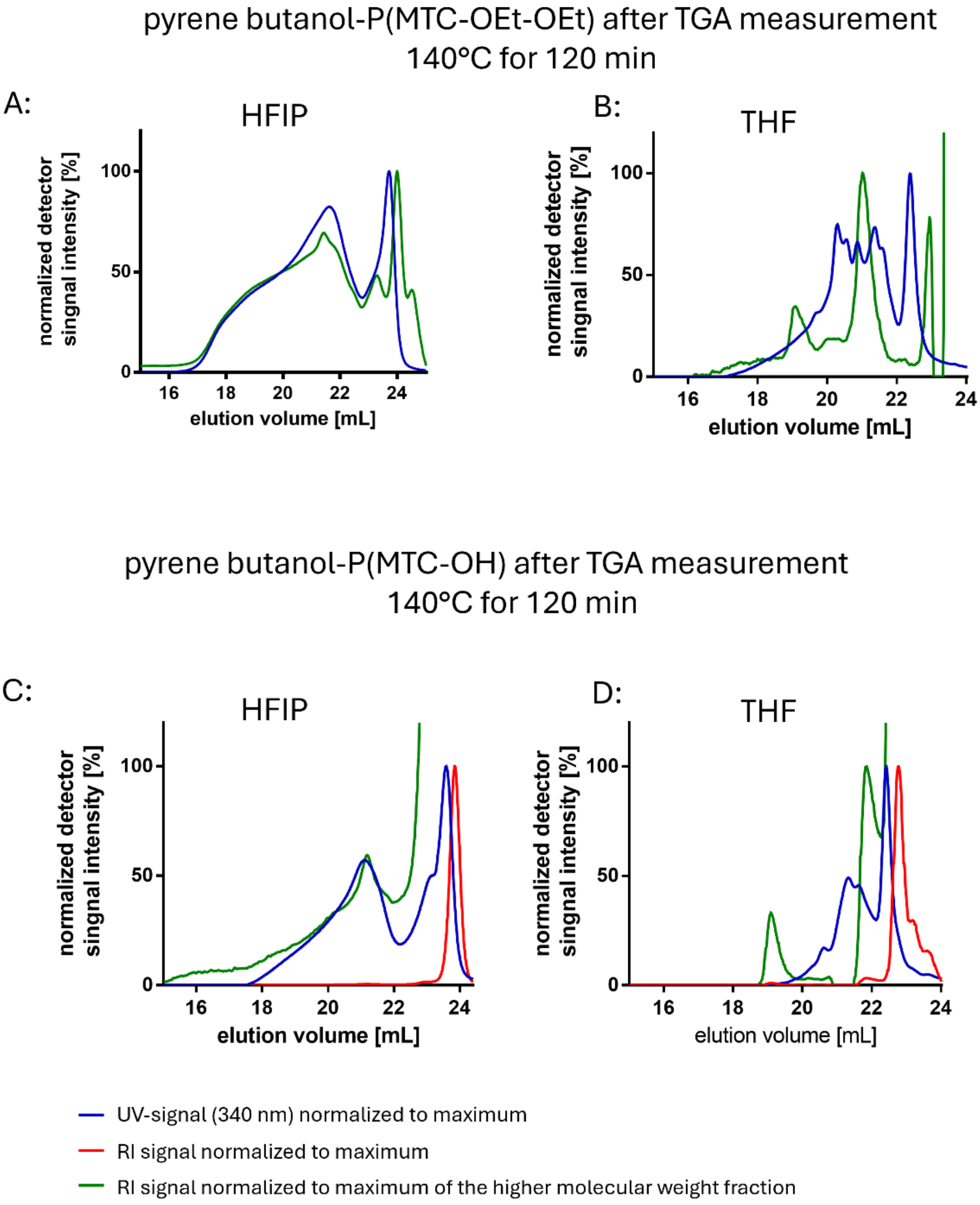

Figure S38: SEC elugrams of the residual polymer after thermogravimetric analysis of pyrene butanol-P(MTC-OEt-OEt)$_{33}$ (A, B) and of pyrene butanol-P(MTC-OH)$_{33}$ (C, D) at 140°C for 120 min. (25-100°C at 10 K/min, 100 -140°C 2K/min). In both solvents we observed insoluble fractions.

^{1}H NMR spectroscopy analysis:

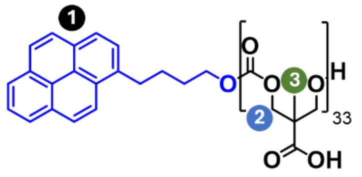

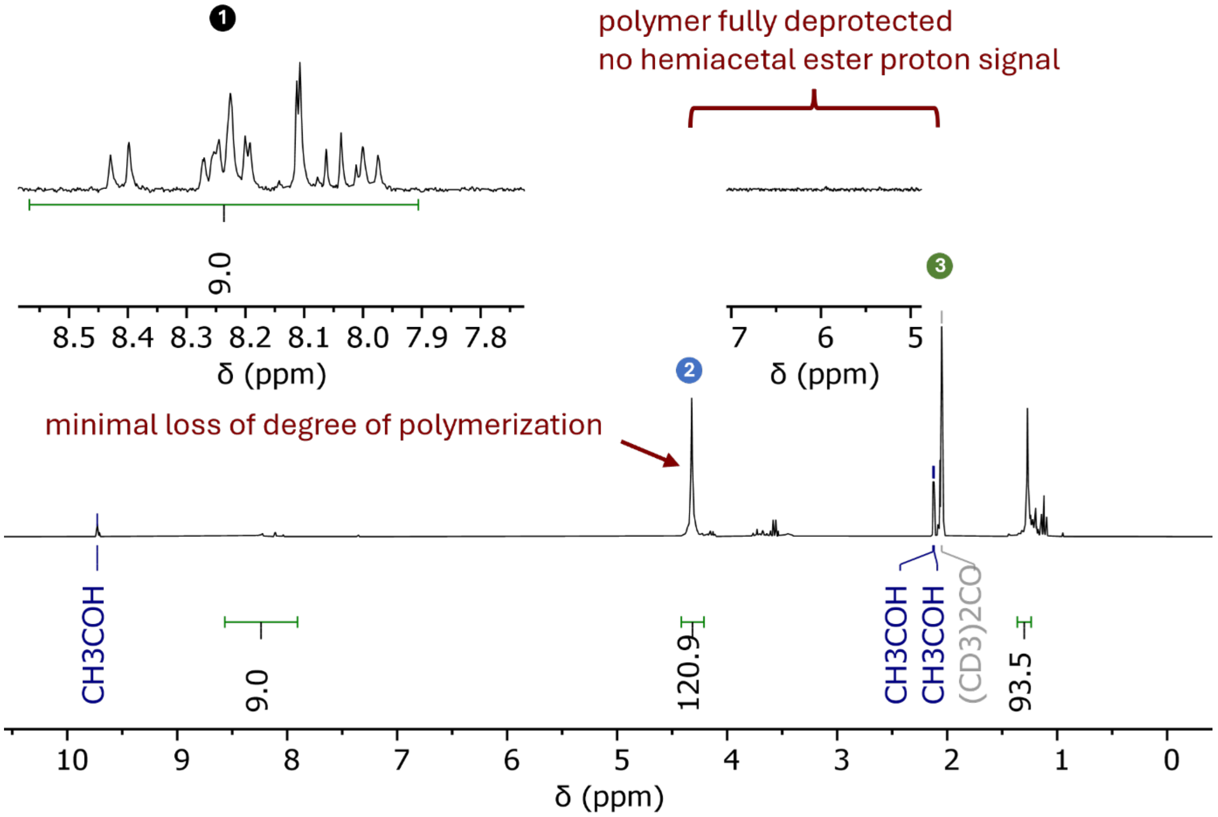

Figure S39: ^{1}H NMR spectrum (300 MHz, DMSO-d_6) of the residual polymer after thermogravimetric analysis of pyrene butanol-P(MTC-OEt-OEt)$_{33}$ at 140°C for 120 min. (25-100°C at 10 K/min, 100 -140°C 2K/min)

Deprotection by acetic acid:

Deprotection by acetic acid. 6.0 mg of pyrene butanol-P(MTC-OEt-OEt)$_{33}$ was dissolved in 500 µL of acetic acid at room temperature. The solution was stirred for 5 h, then all volatile compounds were removed under vacuum, leaving 4.1 mg of the pure deprotected polymer as a white solid. The obtained polymer was characterized by ^{1}H NMR spectroscopy (Figure S40), MALDI-TOF MS analysis (Figure 6D and E) and HFIP SEC (Figure S41).

^{1}H NMR, Figure S40, (300 MHz, acetone-d$_6$) δ 8.45 – 7.78 (m, 9H), 4.32 (s, 132H), 1.29 – 1.23 (m, 108H).

SEC (HFIP) $M_{n\,(RI)}$ ~4 500 g/mol, $Đ_{RI}$ = 1.12 (Figure S41).

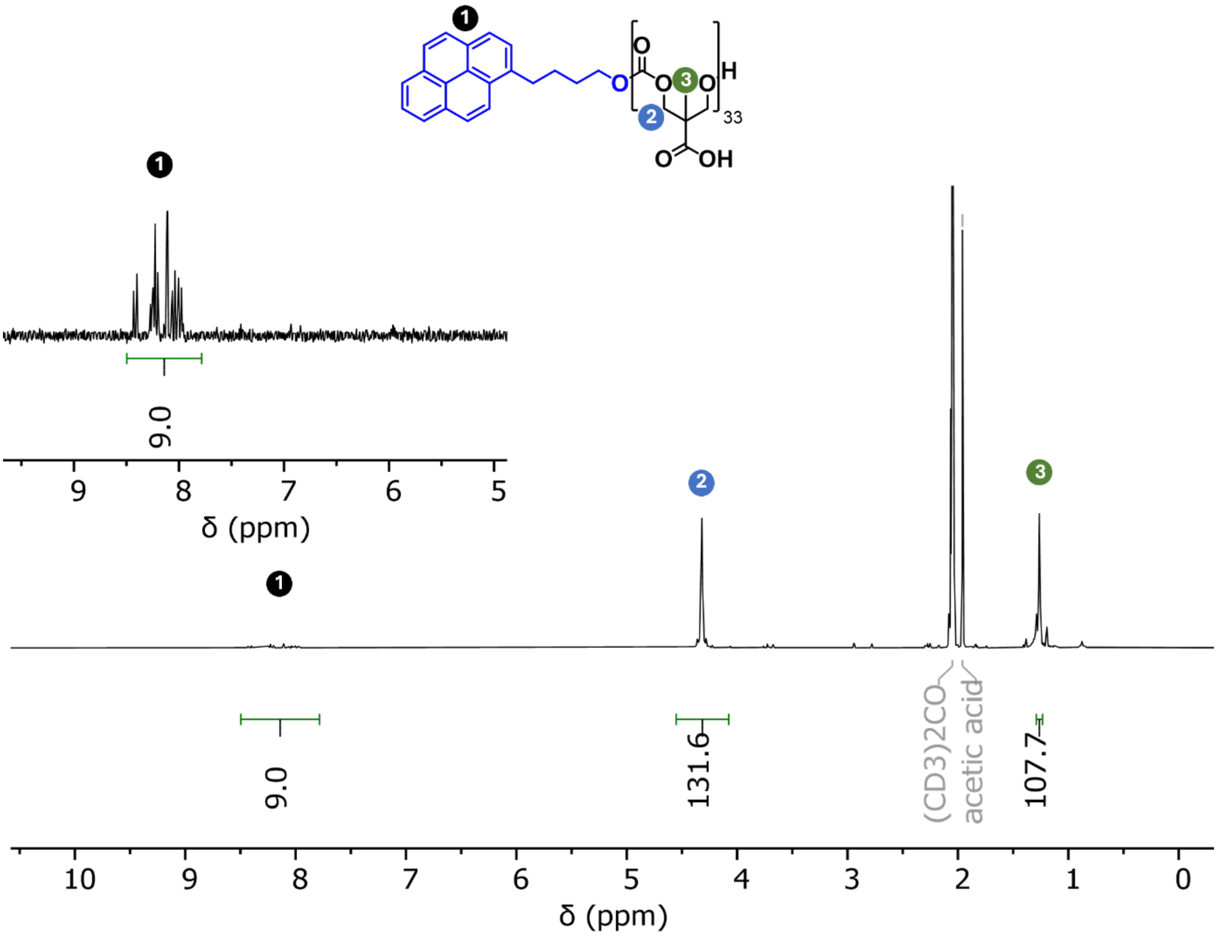

Figure S40: ^{1}H NMR spectrum (400 MHz, acetone-d$_6$) of pyrene butanol-P(MTC-OH)$_{33}$ obtained by deprotection with acetic acid.

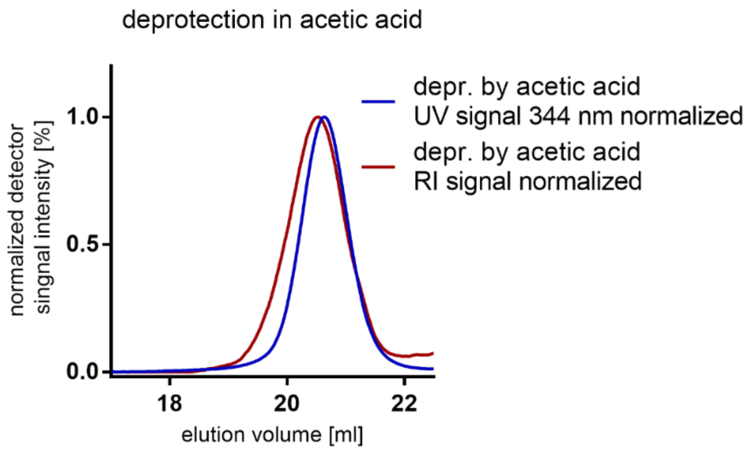

Figure S41: HFIP-SEC elugram of pyrene butanol-P(MTC-OH)$_{33}$ obtained by deprotection with acetic acid.

Table S4: Summary of the polymer characterization obtained through deprotection with acetic acid.

$X_n^{product}$ (NMR)	M_n^{prod} (NMR)	M_n^{prod} (RI-SEC) In HFIP	M_n^{prod} (UV_{344nm}-SEC) In HFIP	$Đ_{UV\text{-}344\ nm}$	$Đ_{RI}$
33	8 000	4 500	4 100	1.10	1.12

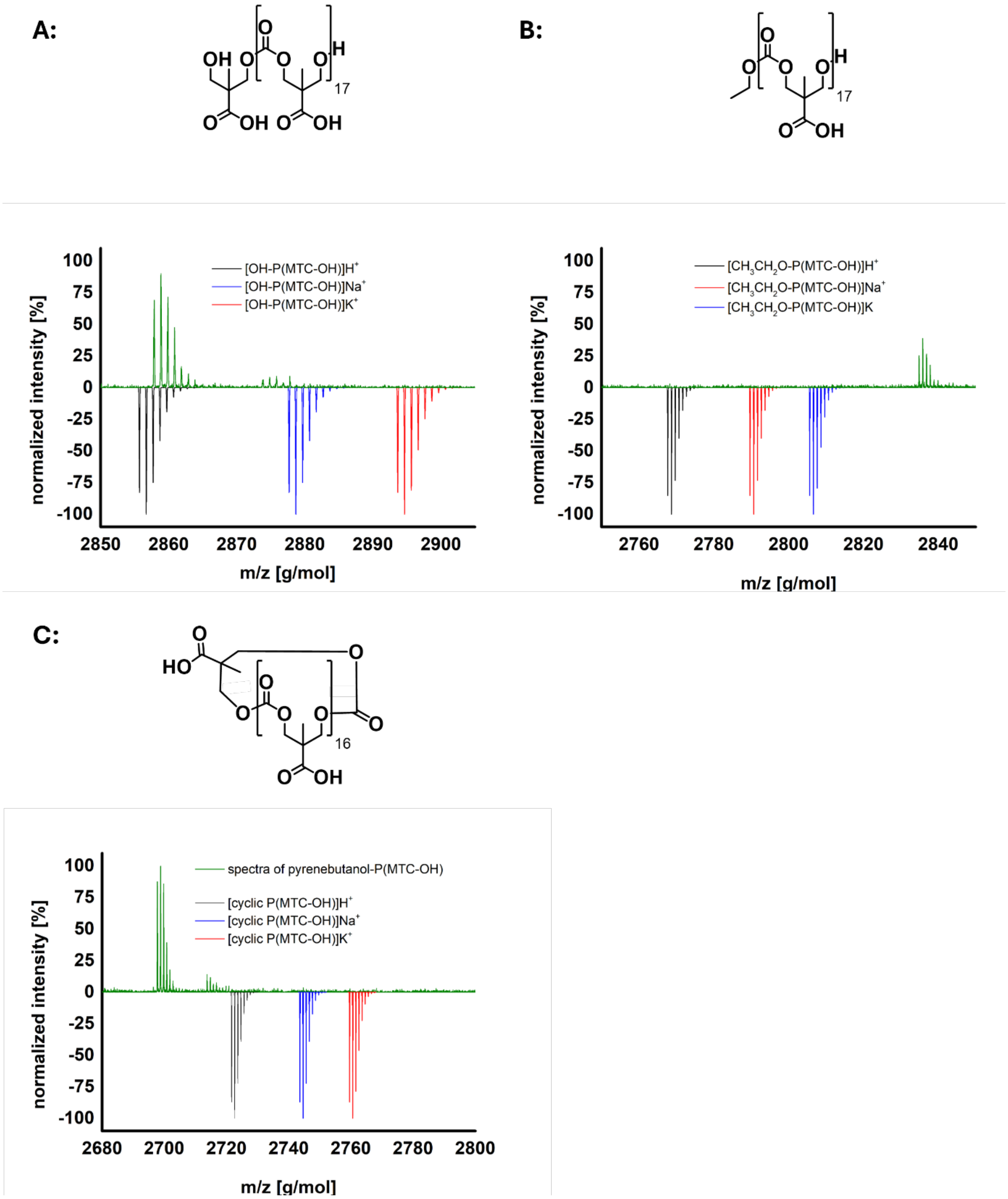

Figure S42: MALDI-TOF MS spectrum analysis: Overlay of the measured spectra of pyrene butanol-P(MTC-OH)$_{33}$ (green, plotted in the positive direction) and simulations of potential side products obtained by (A) initiation by water, (B) initiation by ethanol, (C) cyclic polymer structures by backbiting reactions, which are all absent.

Aqueous Block Copolymer Deprotection:

Aqueous Block Copolymer Deprotection.

The hemiacetal ester deprotection can theoretically be carried out at all pH values; however, since polycarbonates hydrolyze under basic pH conditions, the deprotection should be conducted at neutral or acidic pH values.

Block Copolymer deprotection in pure water (Figure S43).

10 mg mPEG$_{5k}$-*b*-P(MTC-OEt-OEt)$_{11}$ was dissolved in 0.5 mL water and stirred for 5 hours. The solution was freeze-dried yielding 8.85 mg (99 % yield) of pure deprotected mPEG$_{5k}$-*b*-P(MTC-OH)$_{11}$. The deprotected polymer mPEG$_{5k}$-*b*-P(MTC-OH)$_{11}$ is insoluble in THF, therefore SEC trace was performed with HFIP as solvent. **^{1}H NMR**, Figure S44, (300 MHz, DMSO-*d*$_6$) δ 4.18 (s, 44H), 3.51 (s, 475H), 3.24 (s, 3H), 1.17-1.03 (m, 34H). **SEC (HFIP)** $M_{n\ (RI)}$ ~28 500 g/mol, $Đ_{RI}$ = 1.05 (Figure S45).

Degradation of mPEG$_{5k}$-*b*-P(MTC-OEt-OEt)$_{11}$ at neutral pH (Figure 8). ~ 4 mg of mPEG$_{5k}$-*b*-P(MTC-OEt-OEt)$_{11}$ was dissolved in DCM-d$_2$ and analyzed by ^{1}H NMR spectroscopy to confirm the integrity of the hemiacetal ester. After analysis, the DCM was evaporated under reduced pressure, and 0.5 mL of 0.4 M deuterated sodium phosphate buffer (adjusted to neutral pH (according to mQuant pH indicator strips ranging from pH 4.0 – 7.0 and 6.5 to 10.0) was added to the residue, followed by vigorous shaking. Immediately after mixing, the solution was transferred to an NMR tube and analyzed sequentially by ^{1}H NMR spectroscopy.

Block Copolymer deprotection in pure water:

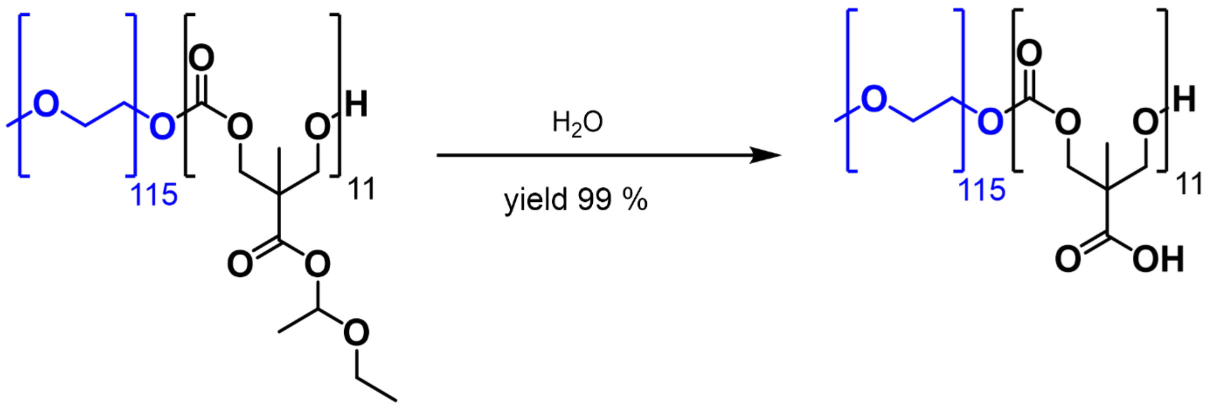

Figure S43: Hemiacetal ester deprotection of mPEG$_{5k}$-*b*-P(MTC-OEt-OEt)$_{11}$ by pure water yielding mPEG$_{5k}$-*b*-P(MTC-OH)$_{11}$

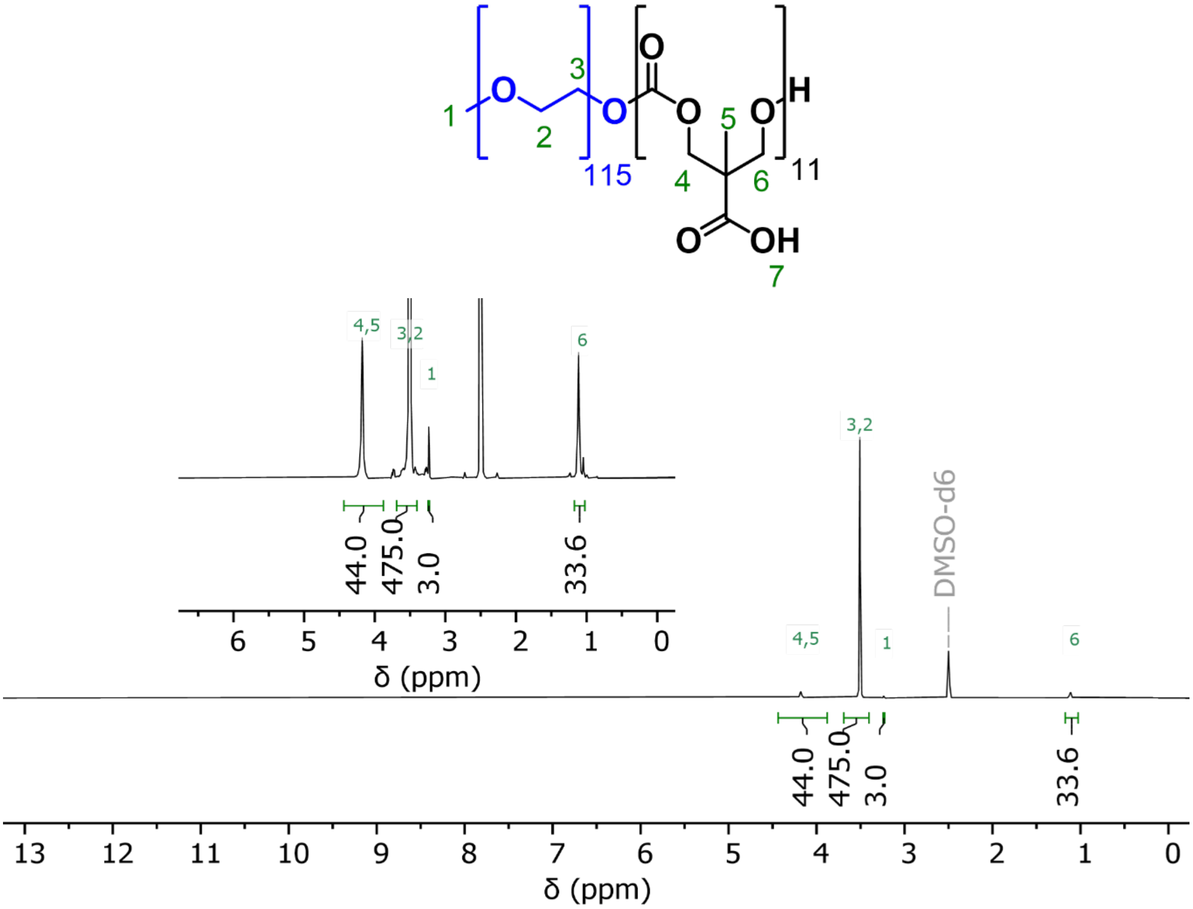

Figure S44: ^{1}H NMR spectrum (300 MHz, DMSO-d_6) of mPEG$_{5k}$-*b*-P(MTC-OH)$_{11}$ obtained by deprotection in pure water.

Table S5: Summary of the polymer characterization of mPEG$_{5k}$-*b*-P(MTC-OH)$_{11}$ obtained by deprotection in pure water.

$\chi_n^{product}$ (NMR)	M_n^{prod} (NMR)	M_n^{prod} (RI-SEC) In HFIP	$Đ_{RI}$
11	7 200	28 500	1.05

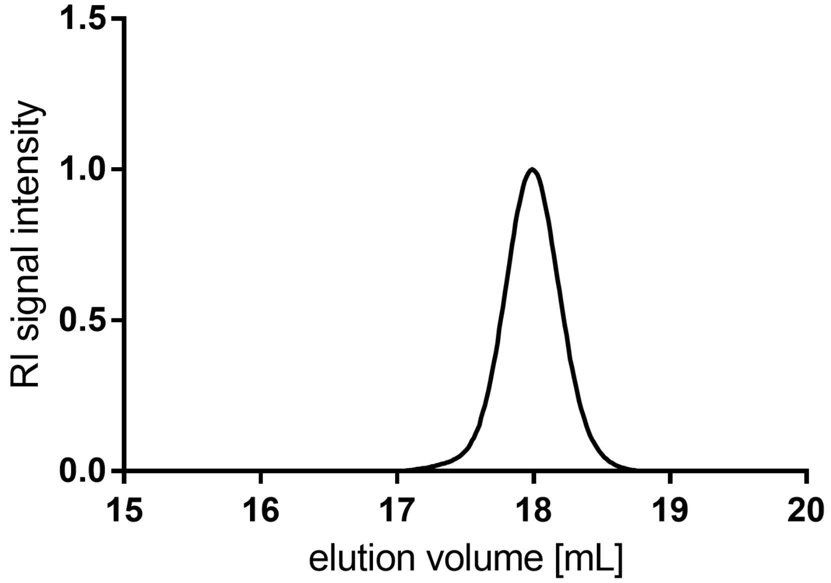

Figure S45: HFIP-SEC trace of mPEG$_{5k}$-*b*-P(MTC-OH)$_{11}$ obtained by deprotection in pure water.

Block Copolymer deprotection in water under acidic conditions:

Block Copolymer deprotection in water under acidic conditions (Figure S46).

A 10 mg sample of mPEG$_{5k}$-*b*-P(MTC-OEt-OEt)$_{11}$ was dissolved in 0.5 mL of water that had been acidified to pH 3 with HCl, and the solution was stirred for 5 hours. The solvent was then removed by freeze drying, resulting in 8.83 mg (99% yield) of pure deprotected polymer. **^{1}H NMR**, Figure S47, (300 MHz, DMSO-*d*$_6$) δ 4.18 (s, 44H), 3.51 (s, 475H)), 1.17-1.03 (m, 33H). **SEC (HFIP)** $M_{n\ (RI)}$ ~27 500 g/mol, $Đ_{RI}$ = 1.07 (Figure S48).

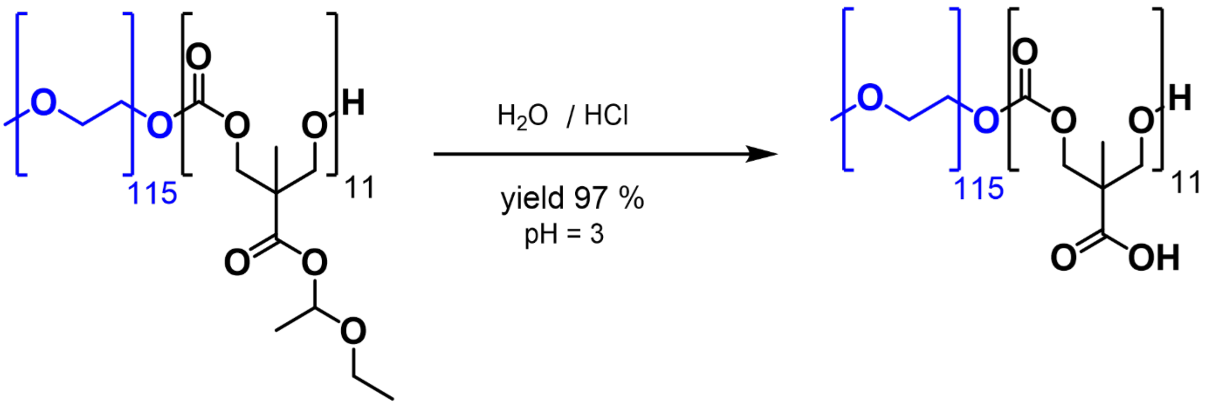

Figure S46: Hemiacetal ester deprotection of mPEG$_{5k}$-*b*-P(MTC-OEt-OEt)$_{14}$ yielding mPEG$_{5k}$-*b*-P(MTC-OH)$_{14}$

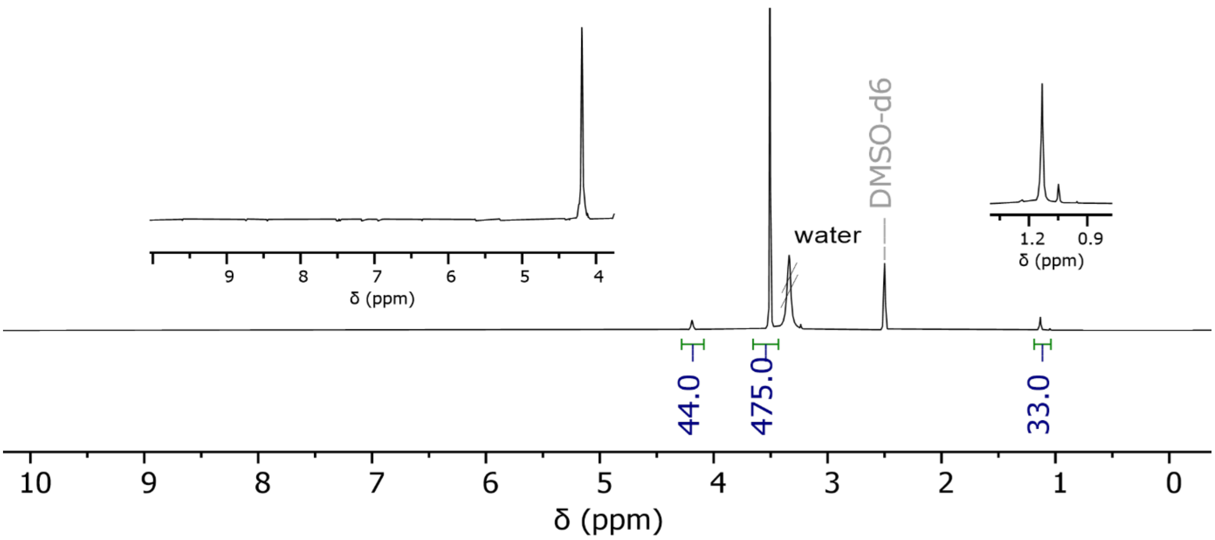

Figure S47: ^{1}H NMR spectrum (300 MHz, DMSO-d6) of mPEG$_{5k}$-*b*-P(MTC-OH)$_{11}$ obtained by deprotection in aqueous HCl solution at pH 3.

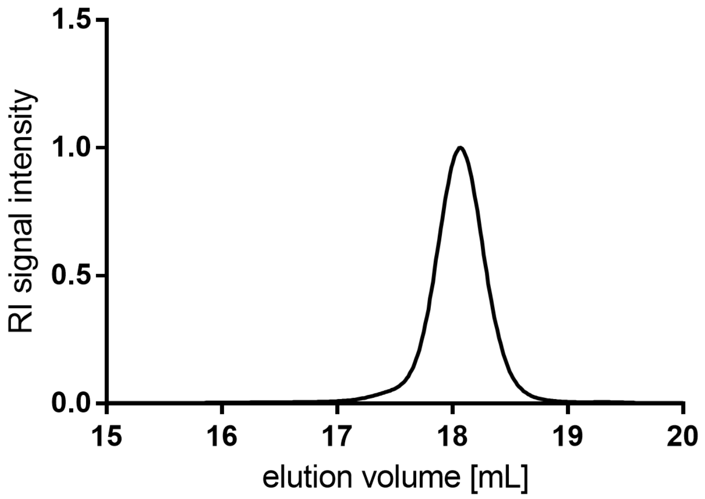

Figure S48:HFIP-SEC trace of mPEG$_{5k}$-*b*-P(MTC-OH)$_{11}$ obtained by deprotection in aqueous HCl solution at pH 3.

Table S6: Summary of the polymer characterization of mPEG$_{5k}$-*b*-P(MTC-OH)$_{11}$ obtained by deprotection in aqueous HCl solution at pH 3.

$X_n^{product}$ (NMR)	M_n^{prod} (NMR)	M_n^{prod} (RI-SEC) In HFIP	$Đ_{RI}$
11	7 200	27 500	1.07

Table S7: Overview of the SEC trace analyses after different deprotection conditions.

	polymer	repeating unit after deprotection ^{1}H NMR	before deprot-ection $PDI_{RI\ THF}$	after deprot-ection $PDI_{RI\ HFIP}$
heat	pyrene butanol-P(MTCO-OEt-OEt)$_{33}$	30	1.12	2.5 (THF)
ethanol	pyrene butanol-P(MTCO-OEt-OEt)$_{33}$	33	1.12	1.13
acetic acid	pyrene butanol-P(MTCO-OEt-OEt)$_{33}$	33	1.12	1.12
water	mPEG$_{5k}$-P(MTCO-OEt-OEt)$_{11}$	11	1.05	1.05
acidic water	mPEG$_{5k}$-P(MTCO-OEt-OEt)$_{11}$	11	1.05	1.07

SI References

(1) Al-Azemi, T. F.; Bisht, K. S. Novel Functional Polycarbonate by Lipase-Catalyzed Ring-Opening Polymerization of 5-Methyl-5-benzyloxycarbonyl-1,3-dioxan-2-one. *Macromolecules* **1999**, *32* (20), 6536–6540. DOI: 10.1021/ma990639r.

(2) Al-Azemi, T. F.; Bisht, K. S. One-step synthesis of polycarbonates bearing pendant carboxyl groups by lipase-catalyzed ring-opening polymerization. *J. Polym. Sci., Part A:Polym. Chem.* **2002**, *40* (9), 1267–1274. DOI: 10.1002/pola.10212.

(3) Pratt, R. C.; Nederberg, F.; Waymouth, R. M.; Hedrick, J. L. Tagging alcohols with cyclic carbonate: a versatile equivalent of (meth)acrylate for ring-opening polymerization. *Chem. Commun.* **2008** (1), 114–116. DOI: 10.1039/b713925j.

(4) Murayama, M.; Sanda, F.; Endo, T. Anionic Ring-Opening Polymerization of a Cyclic Carbonate Having a Norbornene Structure with Amine Initiators. *Macromolecules* **1998**, *31* (3), 919–923. DOI: 10.1021/ma970878j.

(5) Nederberg, F.; Lohmeijer, B. G. G.; Leibfarth, F.; Pratt, R. C.; Choi, J.; Dove, A. P.; Waymouth, R. M.; Hedrick, J. L. Organocatalytic ring opening polymerization of trimethylene carbonate. *Biomacromolecules* **2007**, *8* (1), 153–160. DOI: 10.1021/bm060795n.

(6) Czysch, C.; Medina-Montano, C.; Dal, N.-J. K.; Dinh, T.; Fröder, Y.; Winterwerber, P.; Maxeiner, K.; Räder, H.-J.; Schuppan, D.; Schild, H.; Bros, M.; Biersack, B.; Feranoli, F.; Grabbe, S.; Nuhn, L. End Group Dye-Labeled Polycarbonate Block Copolymers for Micellar (Immuno-)Drug Delivery. *Macromol. Rapid Commun.* **2022**, *43* (12), e2200095. DOI: 10.1002/marc.202200095.

(7) Tan, E. W. P.; Hedrick, J. L.; Arrechea, P. L.; Erdmann, T.; Kiyek, V.; Lottier, S.; Yang, Y. Y.; Park, N. H. Overcoming Barriers in Polycarbonate Synthesis: A Streamlined Approach for the Synthesis of Cyclic Carbonate Monomers. *Macromolecules* **2021**, *54* (4), 1767–1774. DOI: 10.1021/acs.macromol.0c02880.

(8) Hauck, A. V.; Komforth, P.; Erlenbusch, J.; Stickdorn, J.; Radacki, K.; Braunschweig, H.; Besenius, P.; van Herck, S.; Nuhn, L. Aliphatic polycarbonates with acid degradable ketal side groups as multi-pH-responsive immunodrug nanocarriers. *Biomater. Sci.* **2024.** DOI: 10.1039/d4bm00949e.
